# Supplementary material for: MetaboAnalystR 4.0: a unified LC-MS workflow for global metabolomics
Source: Nat Commun. 2024 May 1;15:3675. doi: 10.1038/s41467-024-48009-6 (PMC11063062; doi:10.1038/s41467-024-48009-6)
Supplement: Supplementary file 1 — Supplementary Information [file 41467_2024_48009_MOESM1_ESM.pdf]

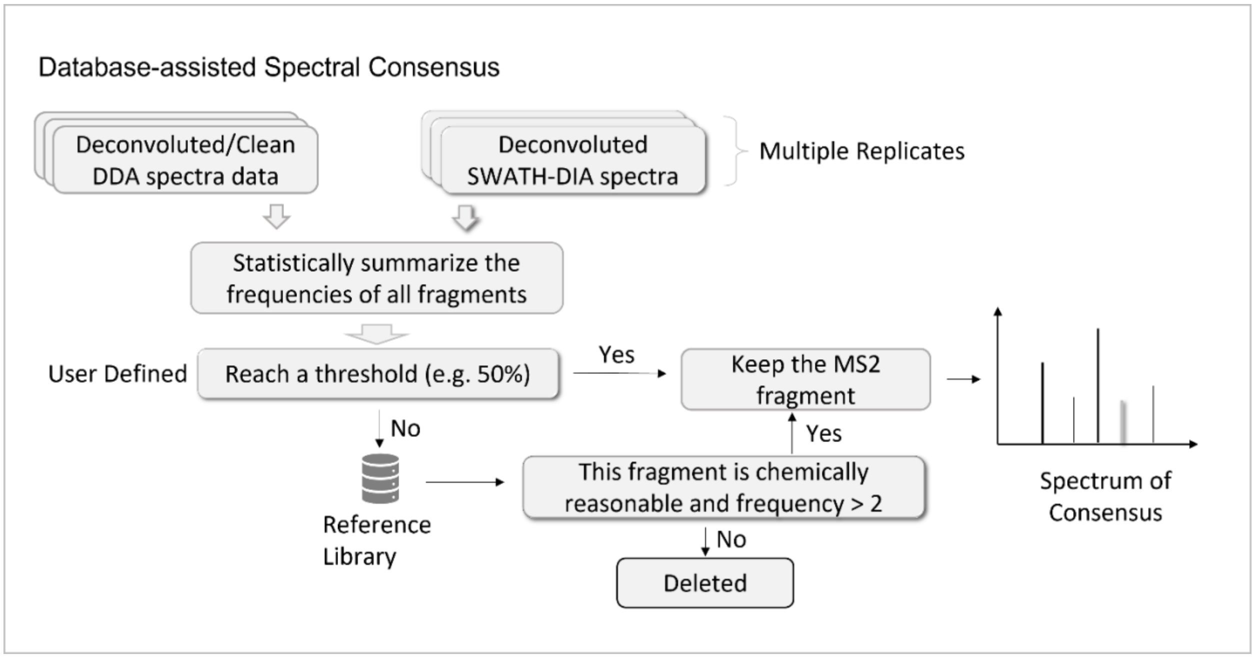

Supplementary Fig. 1. Workflow of spectral consensus for MS2 replicates. The deconvoluted/clean spectra obtained from DDA or SWATH-DIA are summarized by counting the frequency of each fragment. Fragments that meet a user-defined threshold (e.g., 50%) are retained. If the databases-assisted consensus is enabled, fragments that do not meet the threshold are searched against the reference library. All spectra are extracted based on the precursors' information, and if a fragment can be found in any of the extracted spectra, it is considered chemically reasonable. Fragments with a frequency of over 2 are retained, while those with lower frequency are considered noise and deleted.

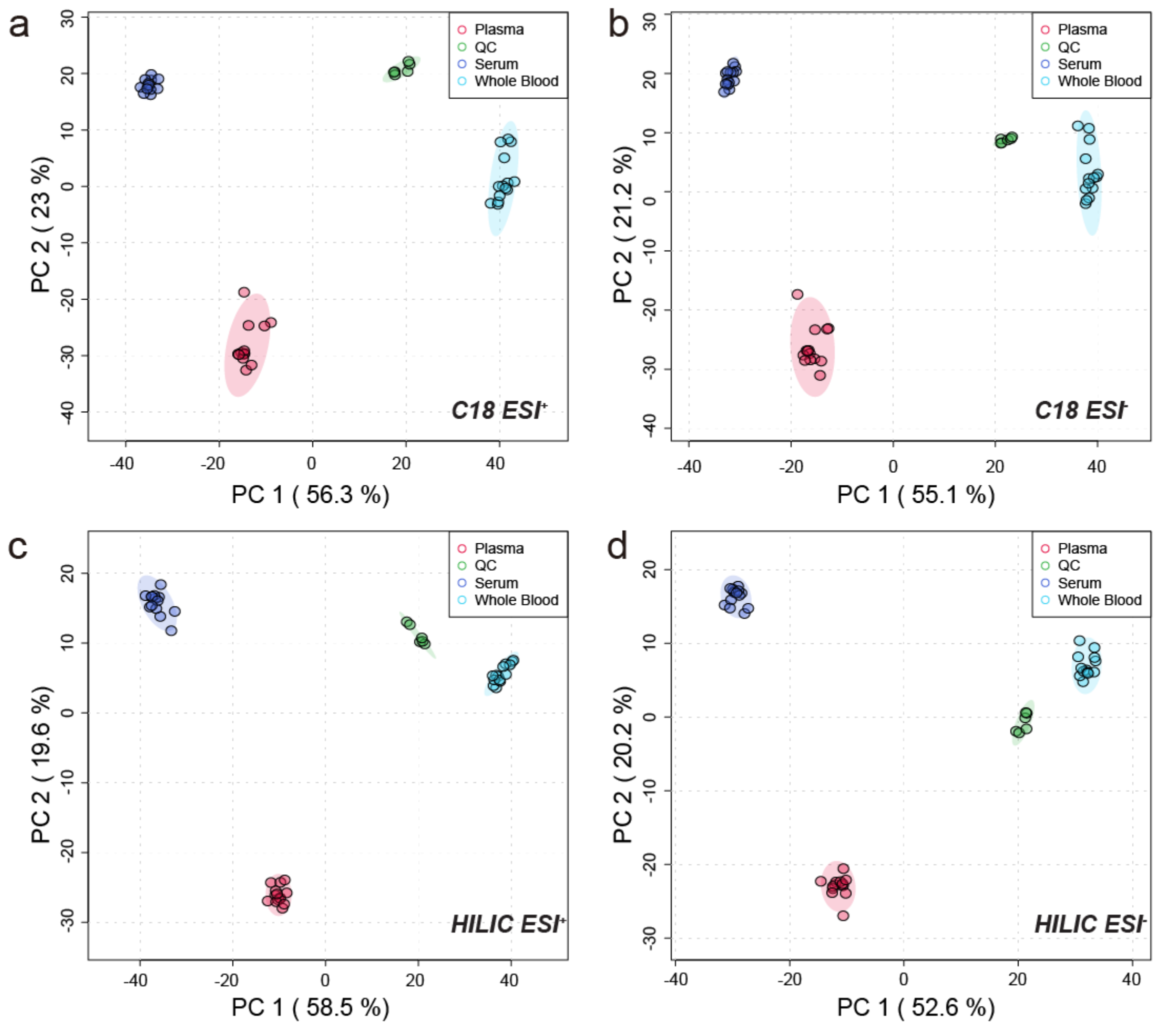

Supplementary Fig. 2. PCA results of the metabolomic profiles of blood samples from four different modes analyzed by MetaboAnalystR. a. C18-ESI^{+} b. C18-ESI^{-}, c. HILIC-ESI^{+} and d. HILIC-ESI^{-}.

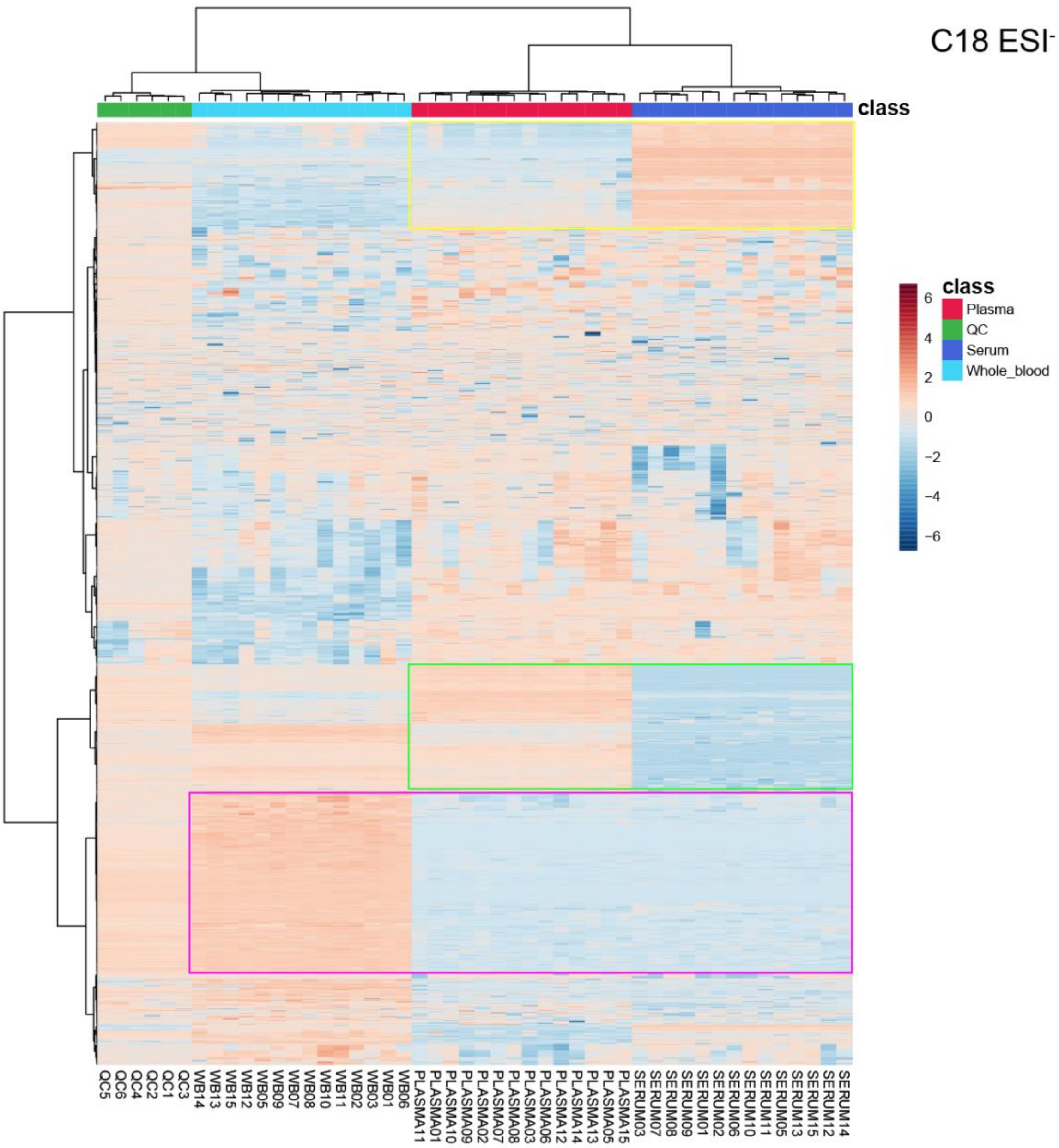

Supplementary Fig. 3. Heatmap of the metabolomic profile of blood samples from C18 ESI$^-$ mode. The heatmap was generated based on the MS1 features detected by MetaboAnalystR. The ruby rectangle highlights unique MS1 features detected in whole blood compared to serum and/or plasma. The green rectangle indicates unique MS1 features detected in plasma compared to serum, while the yellow rectangle highlights unique MS1 features detected in serum compared to plasma.

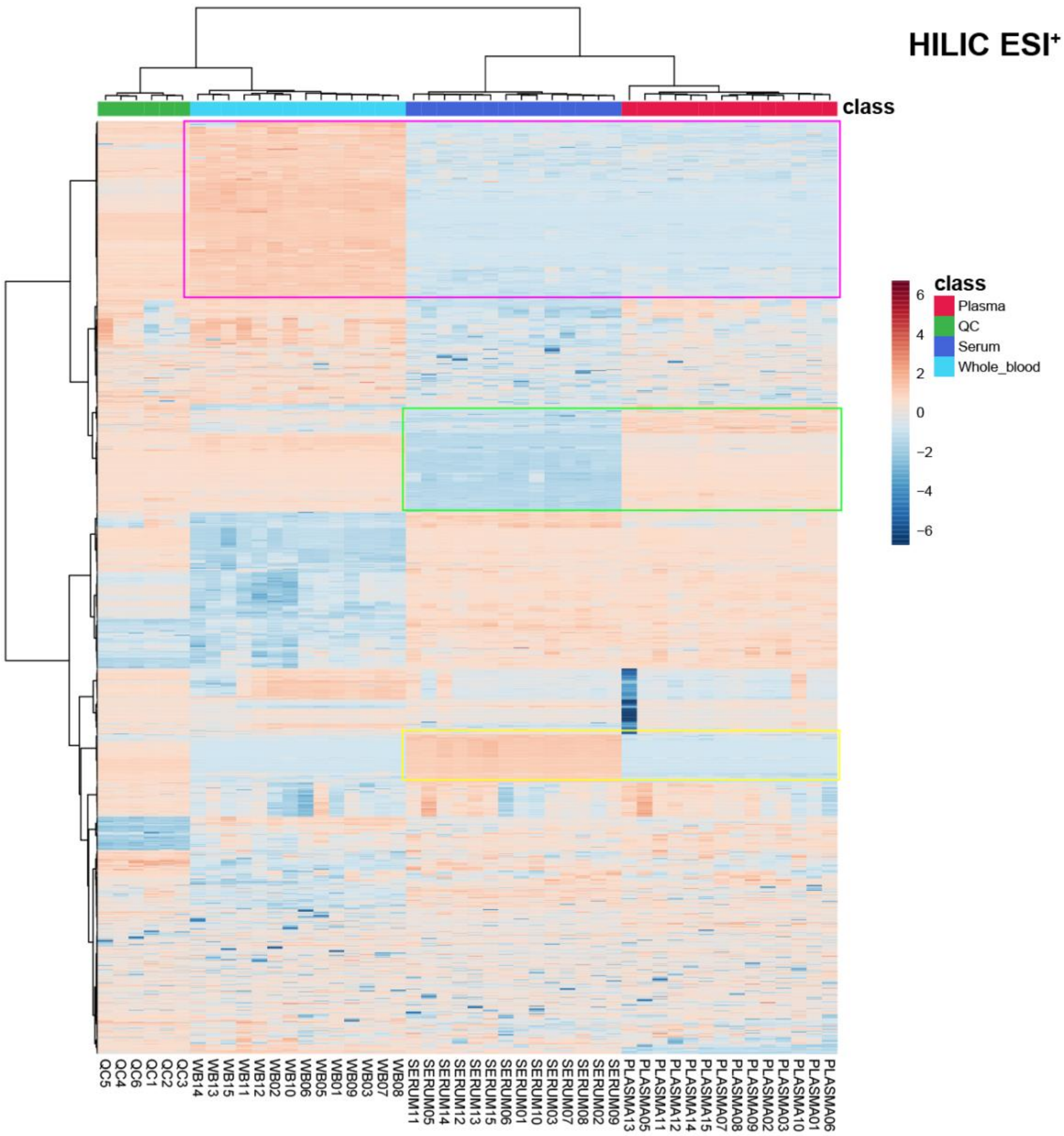

Supplementary Fig. 4. Heatmap of the metabolomic profile of blood samples from HILIC ESI$^+$ mode**.** The heatmap was generated based on the MS1 features detected by MetaboAnalystR. The ruby rectangle highlights unique MS1 features detected in whole blood compared to serum and/or plasma. The green rectangle indicates unique MS1 features detected in plasma compared to serum, while the yellow rectangle highlights unique MS1 features detected in serum compared to plasma.

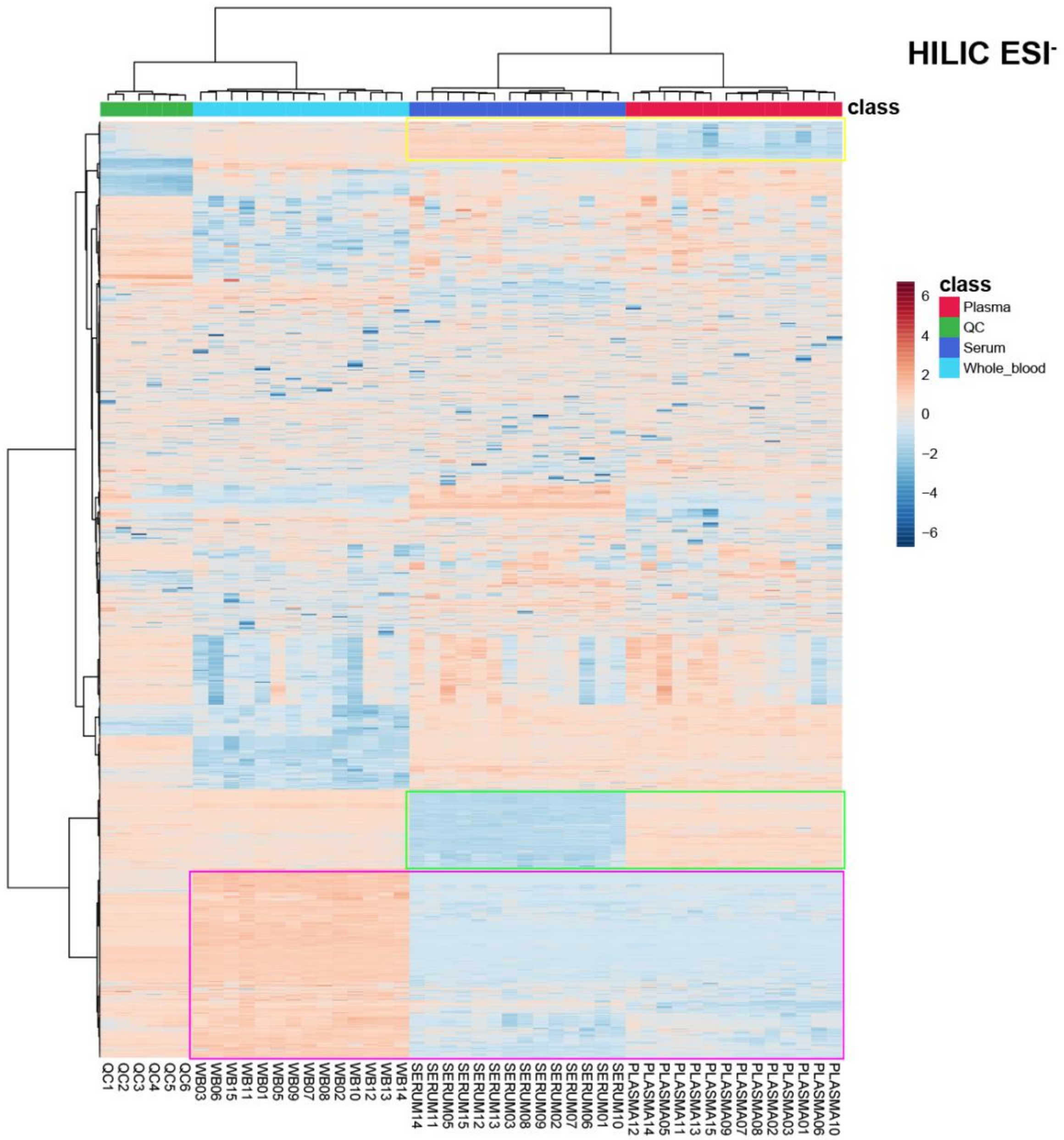

Supplementary Fig. 5. Heatmap of the metabolomic profile of blood samples from HILIC ESI$^-$ mode. The heatmap was generated based on the MS1 features detected by MetaboAnalystR. The ruby rectangle highlights unique MS1 features detected in whole blood compared to serum and/or plasma. The green rectangle indicates unique MS1 features detected in plasma compared to serum, while the yellow rectangle highlights unique MS1 features detected in serum compared to plasma.

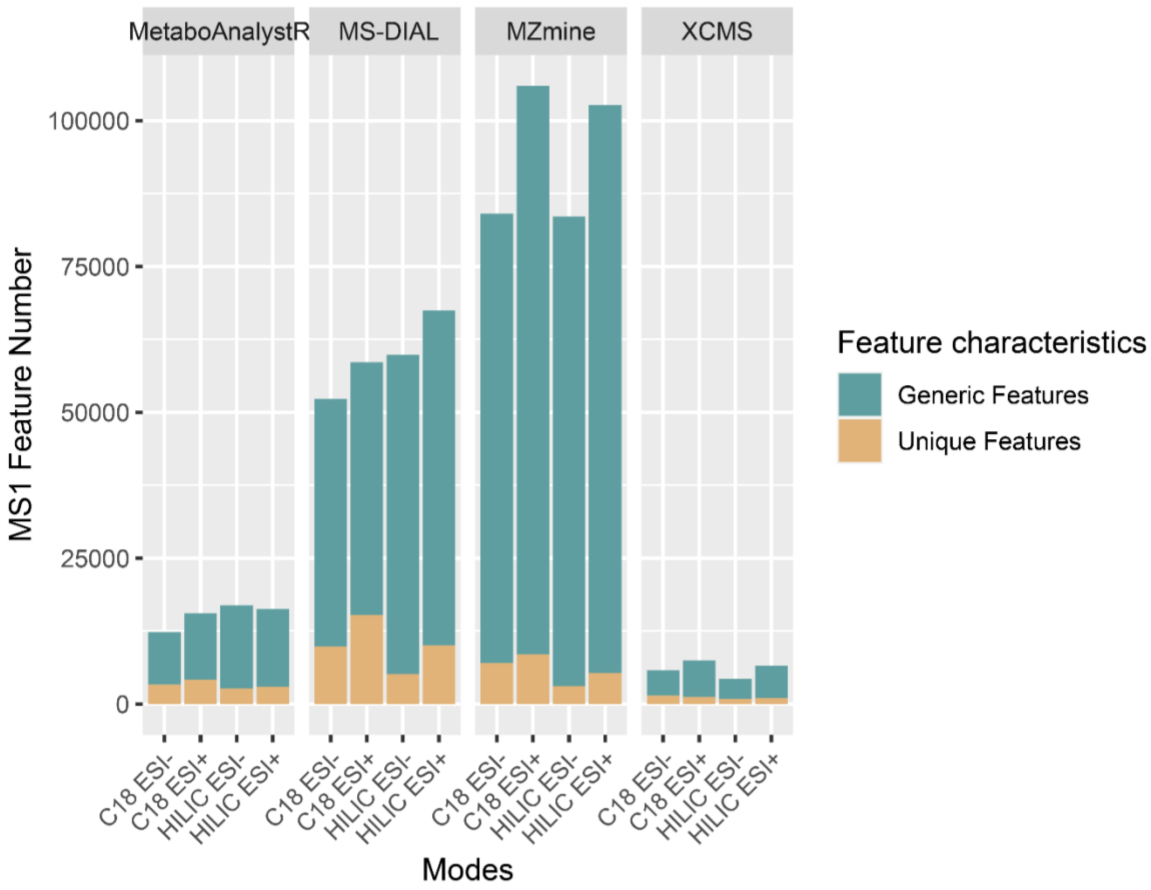

Supplementary Fig. 6. Graphical summary of MS1 features detected by different tools. Features are classified as "Generic Features" or "Unique Features". Generic features can be detected from all blood samples, while unique features are the features detected from a certain blood sample type specifically. The results show that MZmine and MS-DIAL are more sensitive compared to MetaboAnalystR and XCMS, indicating that these tools may be more suitable for detecting features at a trace level.

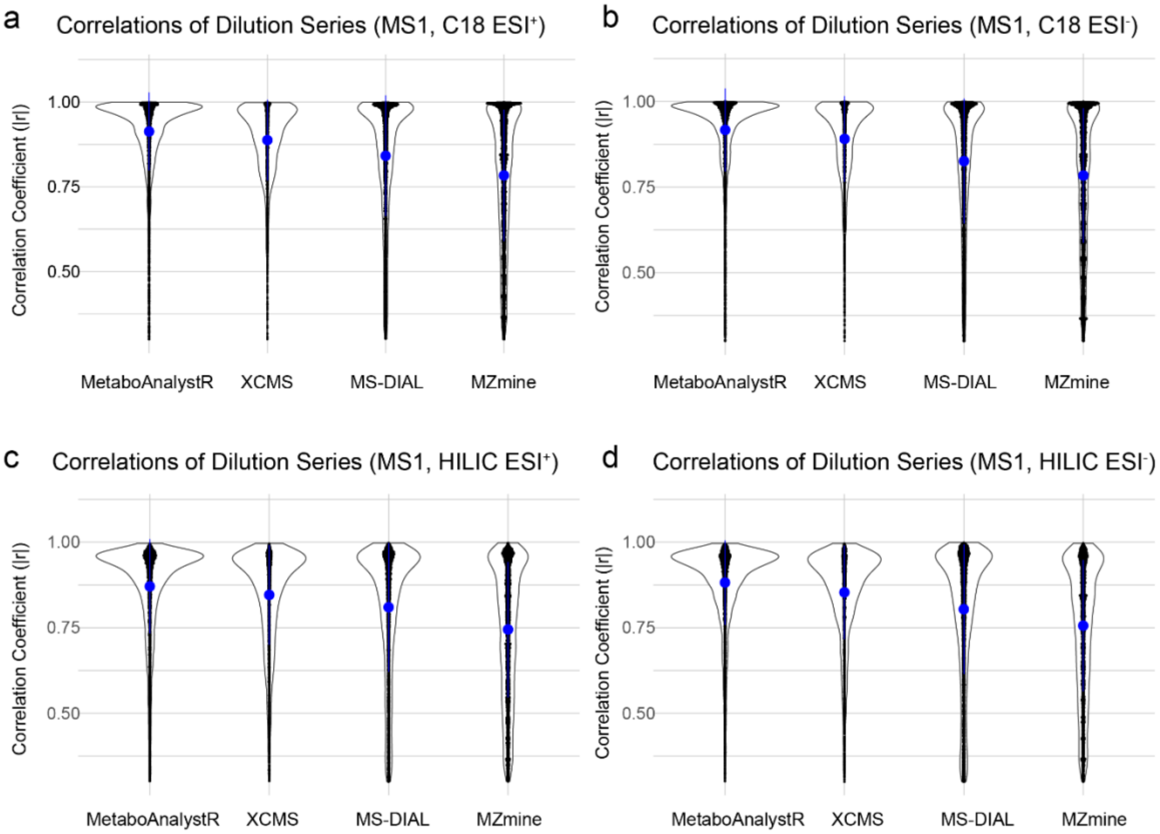

Supplementary Fig. 7. Evaluation of quantitative performance based on serial dilutions. The correlation analyses were performed using MS1 features detected by different tools under four different modes. MetaboAnalystR reported the highest average correlation coefficients with the dilution ratios compared to other tools.

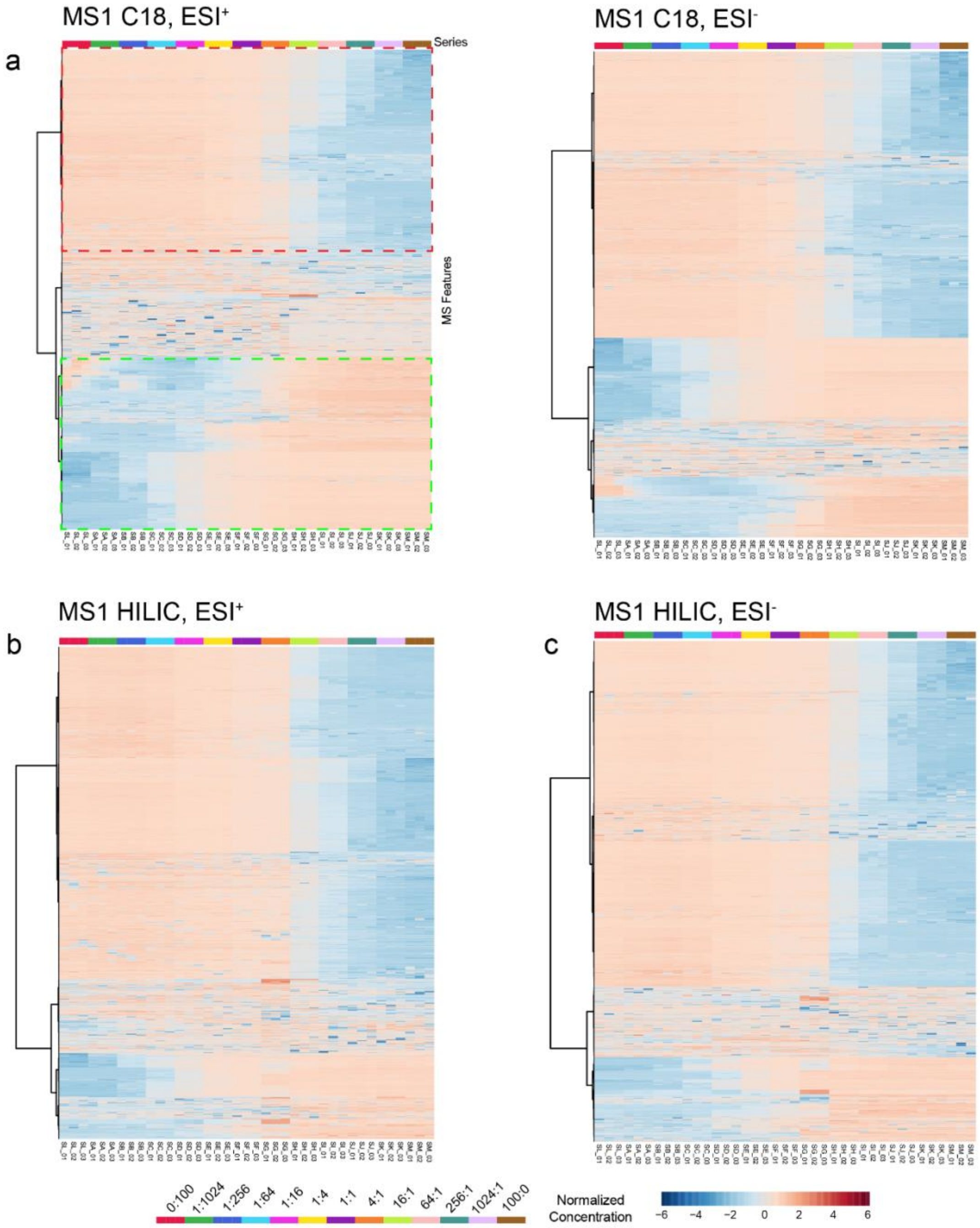

Supplementary Fig. 8. Serial dilution heatmaps. Heatmaps of all MS1 features detected by MetaboAnalystR under different modes (a. C18 ESI$^+$; b. C18 ESI$^-$; c. HILIC ESI$^+$; d. HILIC ESI$^-$). All features have been normalized and clustered, and the samples are not sorted based on the dilution series. Pure urine (100:0) and serum (0:100) samples are also included. Clear serial dilution patterns are evident for all modes. Only the features that are not shared by urine and serum are used for compound identification (the features highlighted with red and green rectangle are considered as not shared).

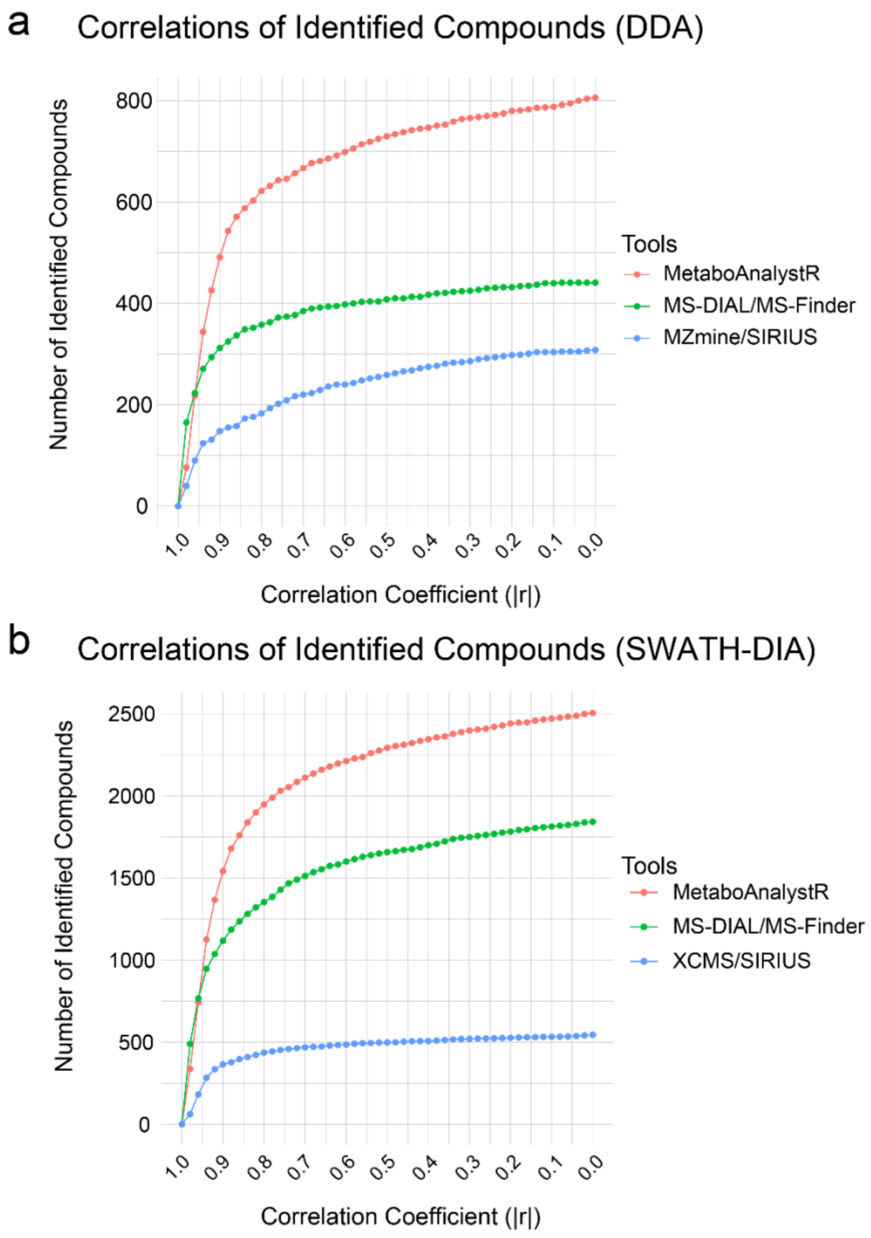

Supplementary Fig. 9. Evaluation of qualitative performance based on serial dilutions. a. Relationships between numbers of identified compounds from DDA mode and correlation coefficient cut-offs. b. Relationships between numbers of identified compounds from SWATH-DIA mode and correlation coefficient cut-offs.

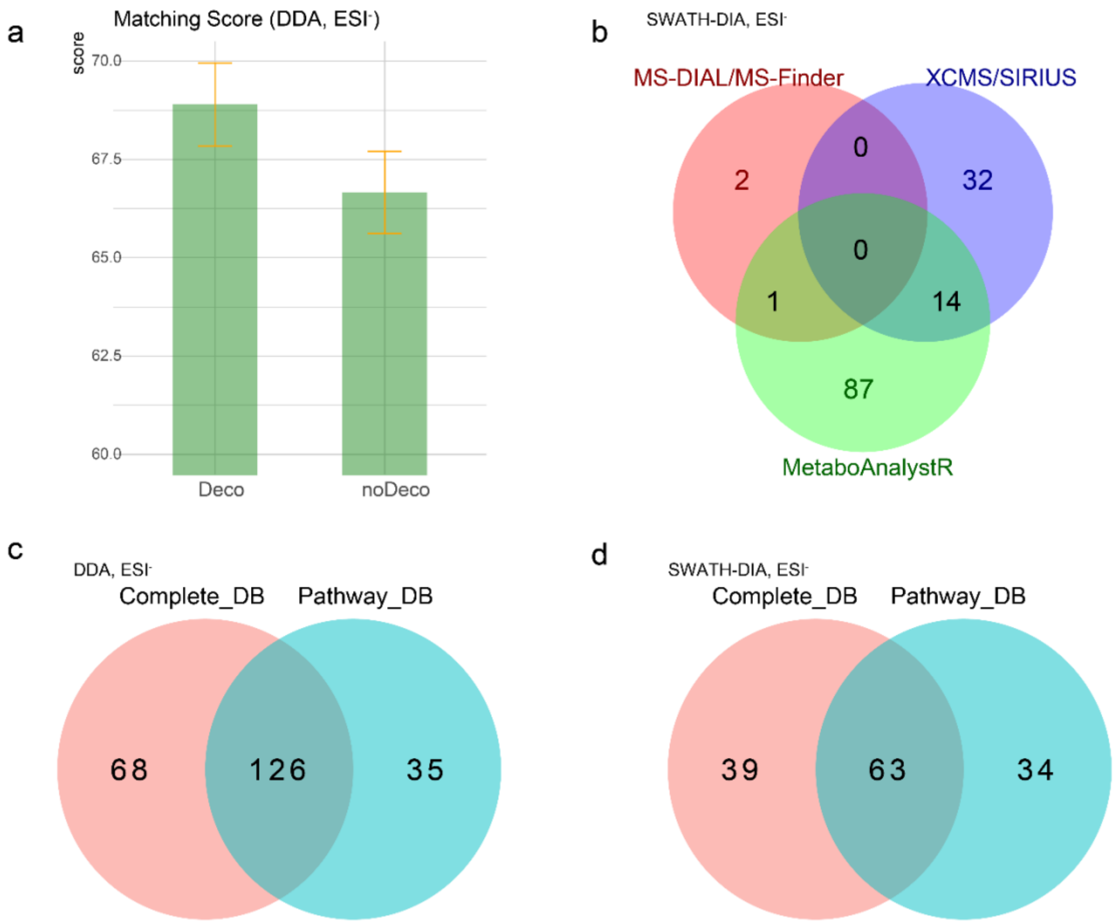

Supplementary Fig. 10. Analysis results based on the complex standards mixture under ESI$^-$ mode. a. Comparison of matching scores of DDA between using deconvolution (deco) and without deconvolution (noDeco). The deconvolution significantly improved the matching scores of chemical candidates. n = 90 independent compounds, means ± SEM; paired one-tailed Student's t-test (**, significant, p = 1.1e-10, no adjustment). b. Venn Diagram of compounds identified from the complex standard mixture by different tools (SWATH-DIA). Performance evaluation of compound identification with different reference libraries by MetaboAnalystR for DDA (c) and SWATH-DIA (d). The majority of the compounds identified with different databases are shared. The pathway library could also be used to find some unique compounds that were masked by false positives from the complete reference library.

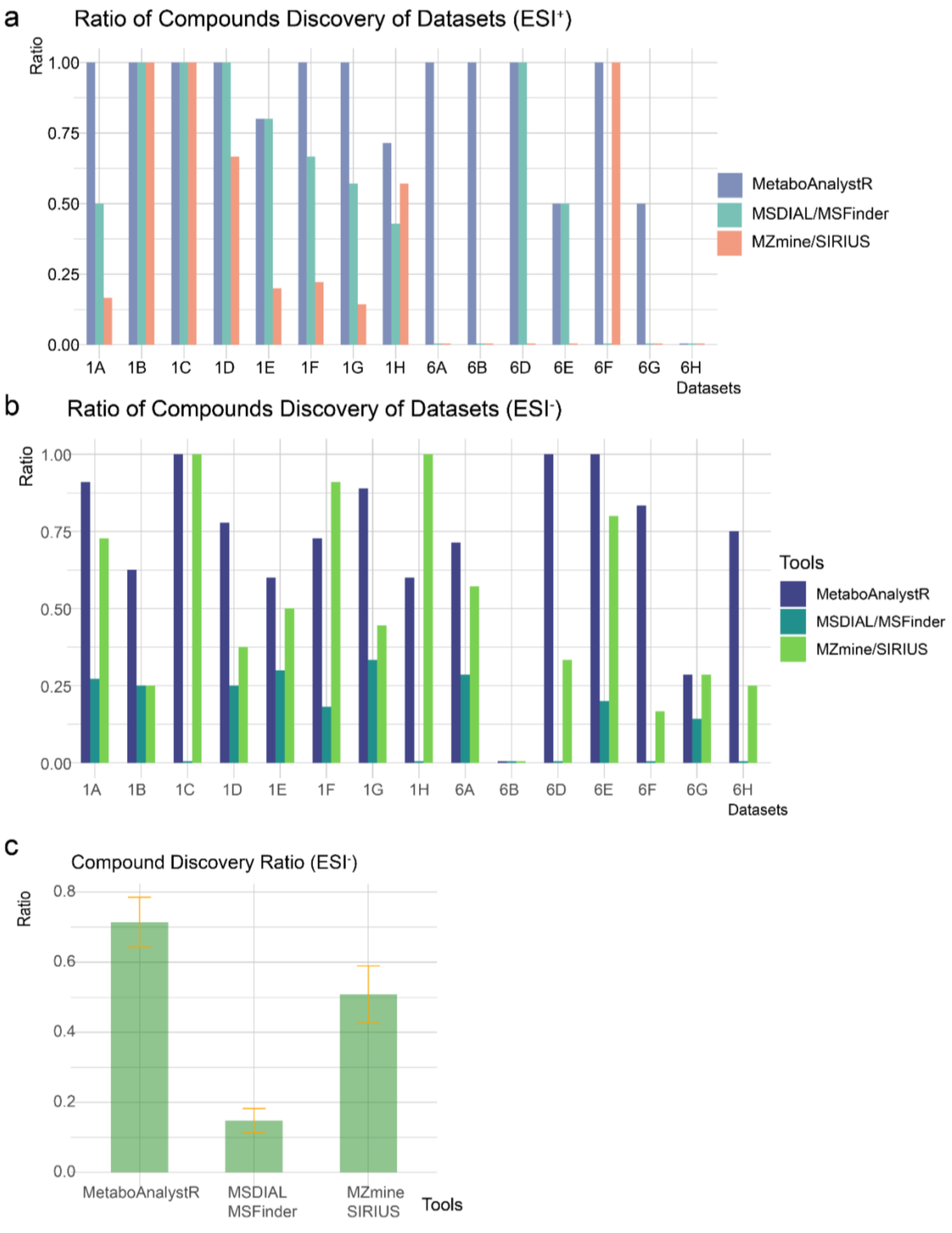

Supplementary Fig. 11. Validation results of MetaboAnalystR with simple standards mixtures. a. Compound discovery ratio in three workflows (ESI^{+}). For all samples, MetaboAnalystR could detect the highest ratio of compounds as the top first candidate. b. Compound discovery ratio in three workflows (ESI^{-}). For all samples, MetaboAnalystR detected the highest ratio of compounds

as the top first candidate. c. Statistical analysis of the compound discovery results. Compared to the other two workflows, MetaboAnalystR reported significantly higher compound discovery ratio. n = 15 independent datasets, means ± SEM; unpaired one-tailed Student's t-test without adjustment (MetaboAnalystR vs. MS-DIAL/MS-Finder, significant, *p*=2.8e-7; MetaboAnalystR vs. MZmine/SIRIUS, significant, *p*=0.033; MS-DIAL/MS-Finder vs. MZmine/SIRIUS, unsignificant, *p*=0.00033).

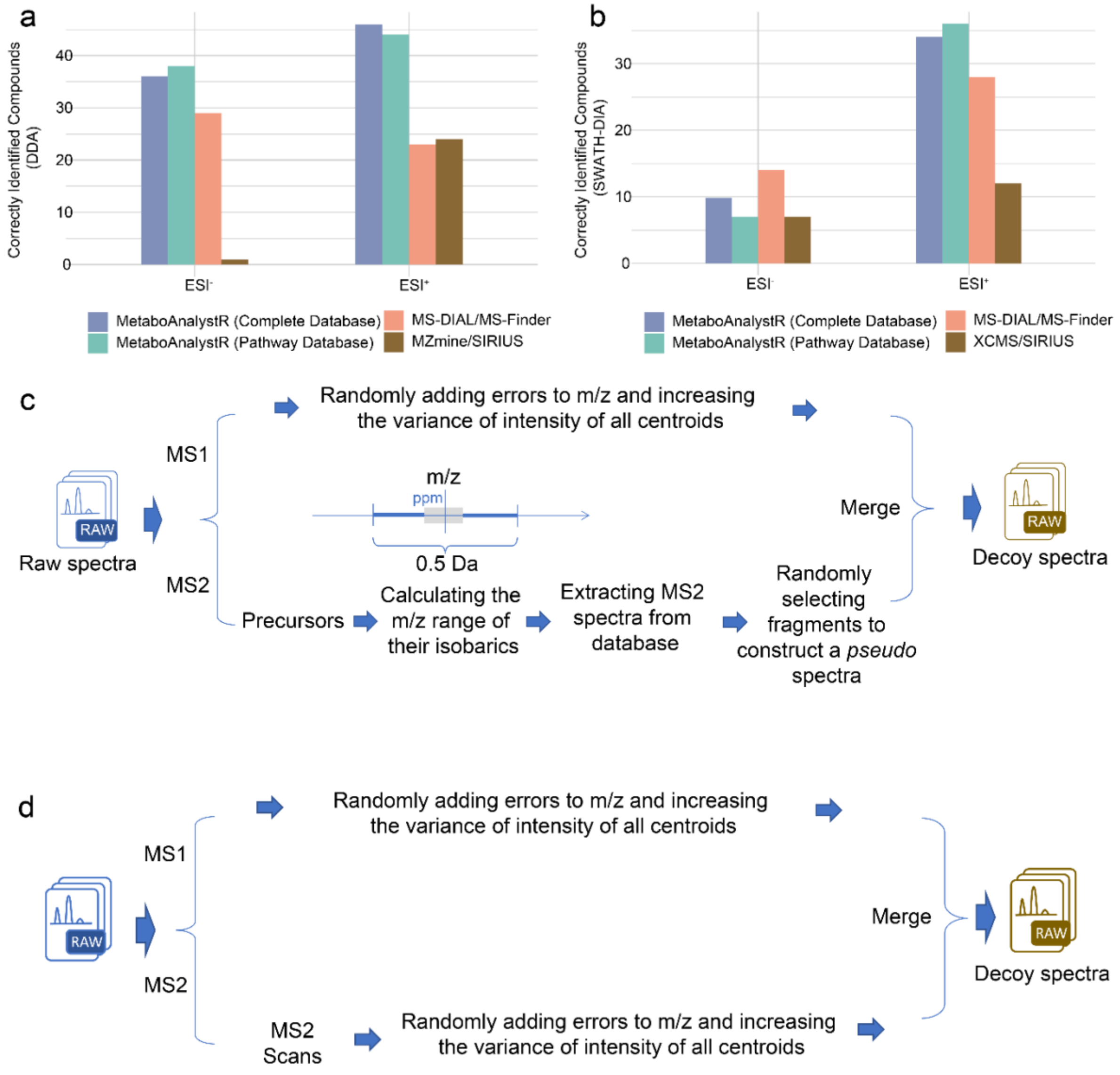

Supplementary Fig. 12. Validation of MetaboAnalystR with standards mixtures. a. Statistics of correctly identified compounds from DDA dataset. b. Statistics of correctly identified compounds from SWATH-DIA dataset. c. Workflow to generate decoy spectra data (DDA). d. Workflow to generate decoy spectra data (SWATH-DIA). Raw spectra data is initially split into MS1 and MS2. For signals from the MS1 level, mass errors (10~30 ppm) for m/z values and variance for

intensities were randomly added, while retention time information is retained in its original status. For MS2 data, the original SWATH windows and cycles are retained, while MS2 spectra were modified by adding mass errors and variance for all MS2 centroids. Finally, all modified MS1 and MS2 scans were merged into decoy spectra data. A total of 18 decoy spectra datasets were generated for both ESI^+ and ESI^-.

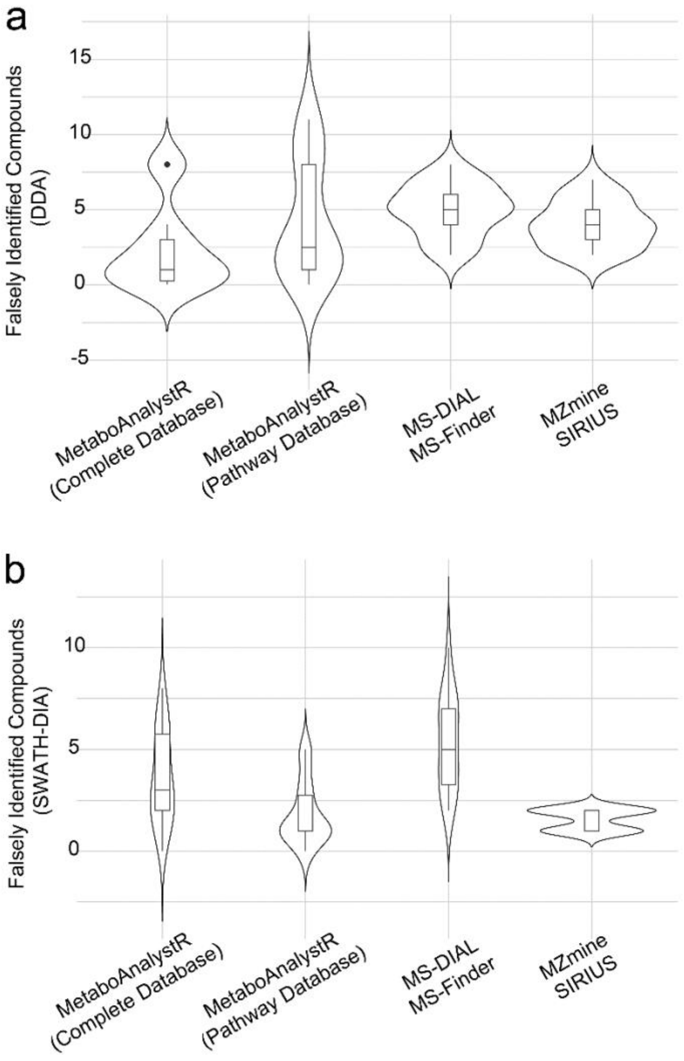

Supplementary Fig. 13. Summary of falsely identified compounds in ESI^+ mode. a. Falsely identified compounds from decoy spectra data (DDA, ESI^+). b. Falsely identified compounds from decoy spectra data (SWATH-DIA, ESI^+). a,b n=18 independent samples used for evaluation. Box plots indicate median, 25th and 75th percentiles (middle line, Q1 and Q3 within the box, respectively), using 1.5x interquartile range (IQR) whiskers and outliers. There is only one outlier point. n = 18 independent samples.

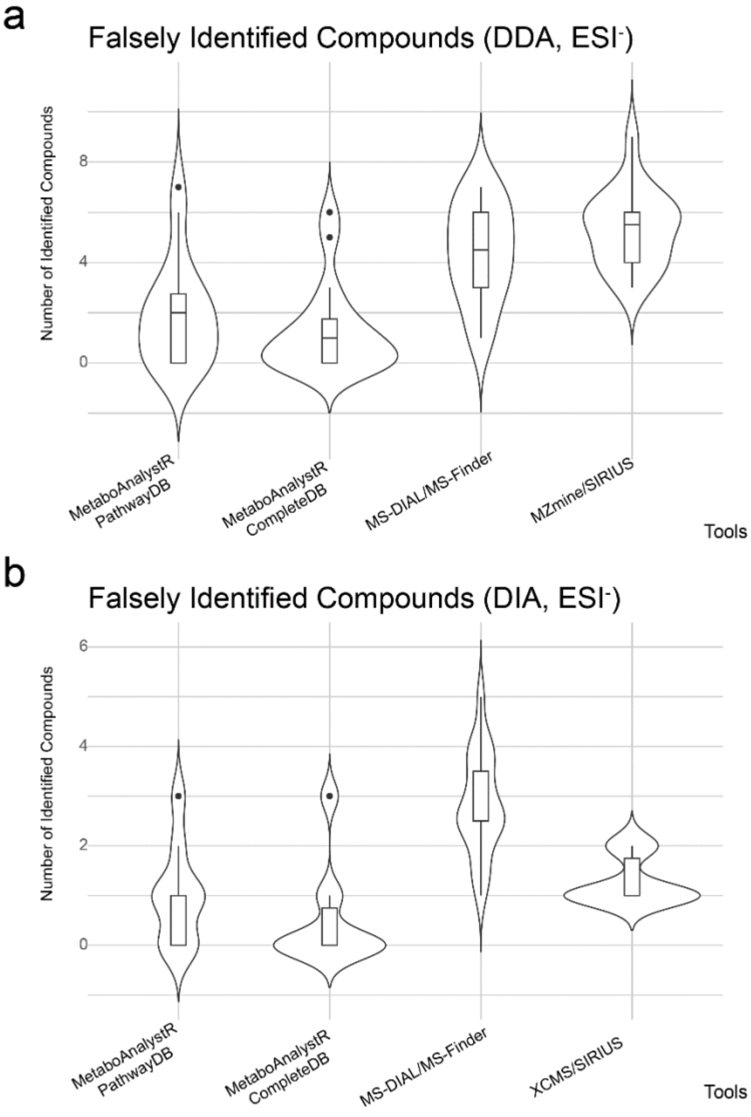

Supplementary Fig. 14. Summary of falsely identified compounds in ESI$^-$ mode. a. falsely identified compounds from decoy spectra data (DDA) are compared between different tools. MetaboAnalystR did not significantly increase the false identification with any reference library, outperforming other tools. b. falsely identified compounds from decoy spectra data (SWATH-DIA) are compared between MetaboAnalystR, XCMS/SIRIUS, and MS-DIAL/MS-Finder workflows. MetaboAnalystR did not significantly increase false identification compared to XCMS/SIRIUS. However, MetaboAnalystR had a significantly lower false identification number compared to MS-DIAL/MS-Finder workflow. a.b Box plots indicate median, 25th and 75th percentiles (middle line, Q1 and Q3 within the box, respectively), using 1.5x interquartile range (IQR) whiskers and outliers. n = 18 independent samples.

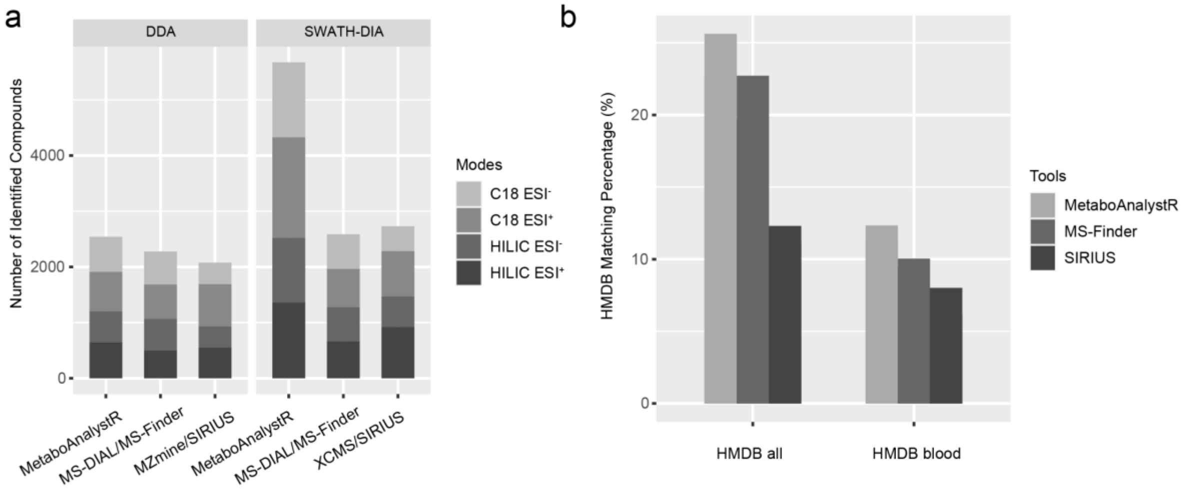

Supplementary Fig. 15. Comparison of identification performance from different blood samples. a. Summary of all compounds identified by different tools. b. HMDB matching percentages for compounds identified by different tools. To evaluate the validity of all chemical identifications, we compared the compounds identified from different tools with HMDB database and HMDB blood database. Compounds identified by MetaboAnalystR had the highest percentage (and absolute number) of compounds matched into databases compared to other tools.

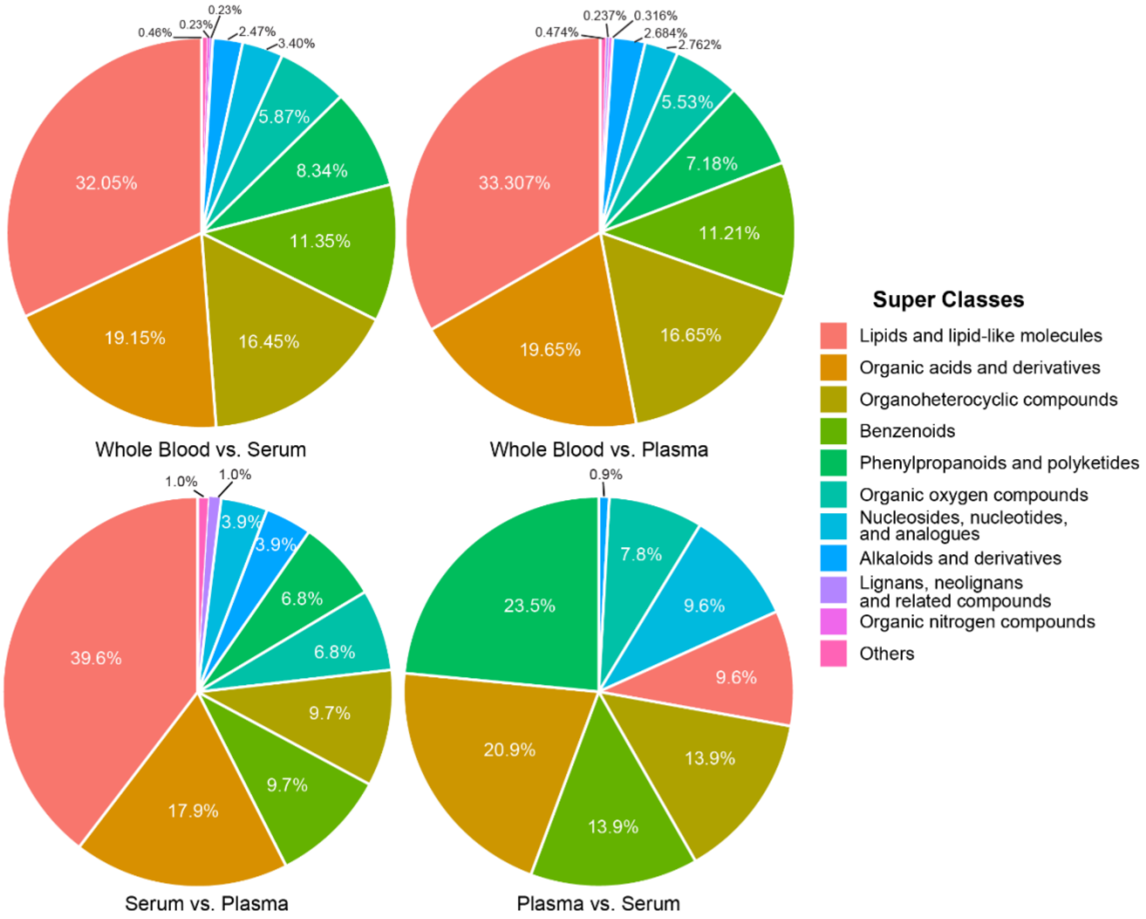

Supplementary Fig. 16. Summary of chemical classification of identified compounds from MetaboAnalystR. All unique features from **Fig. 3d** and Supplementary **Fig. 3-5** were targeted for compound identification. More lipids, organic acids, and organic heterocyclic compounds were identified in whole blood compared to serum and plasma. More lipids, organic acids, and benzenoids were identified in serum compared to plasma. Conversely, more phenylpropanoids, organic heterocyclic and benzenoids were identified in plasma samples compared to serum.

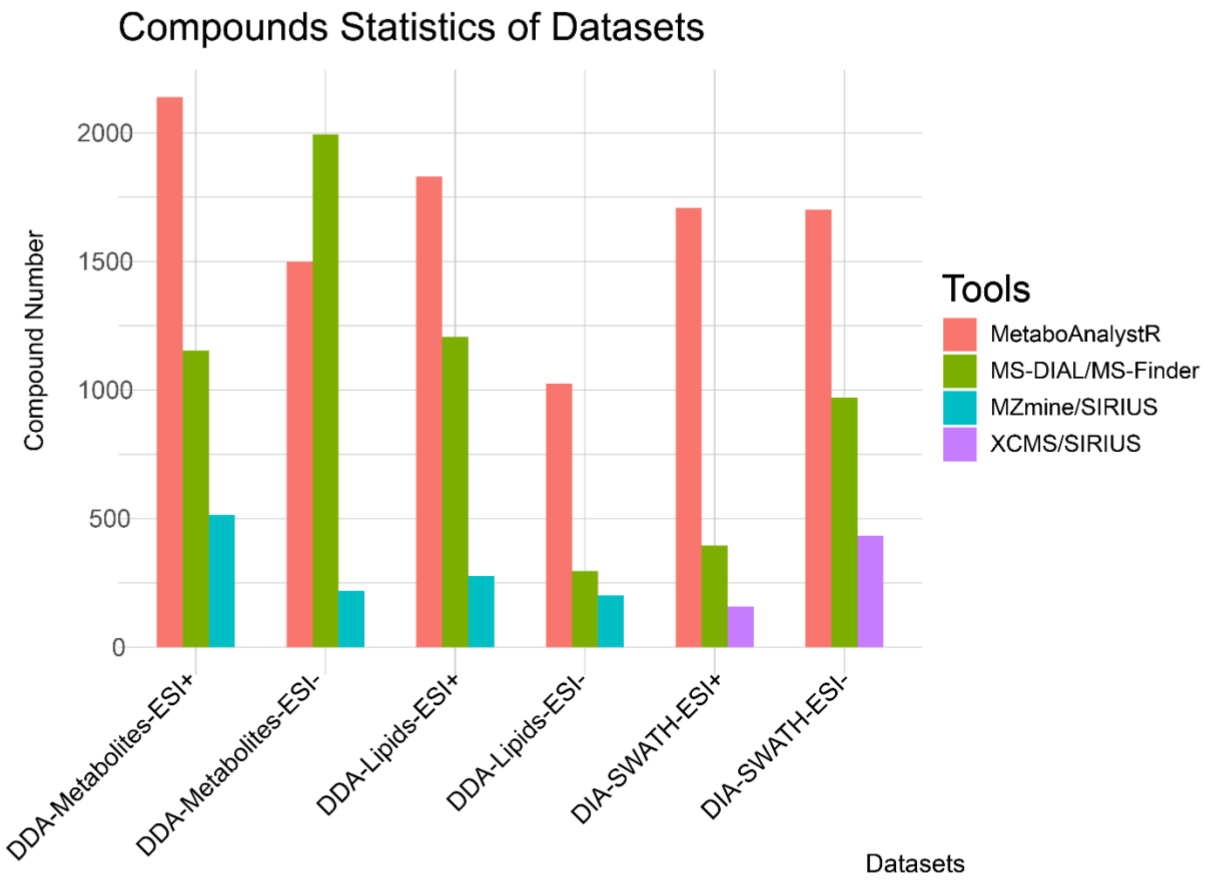

Supplementary Fig. 17. Statics of compound identification by different tools from clinical metabolomics datasets. MetaboAnalystR identified the highest number of compounds except for the DDA dataset in ESI^- mode.

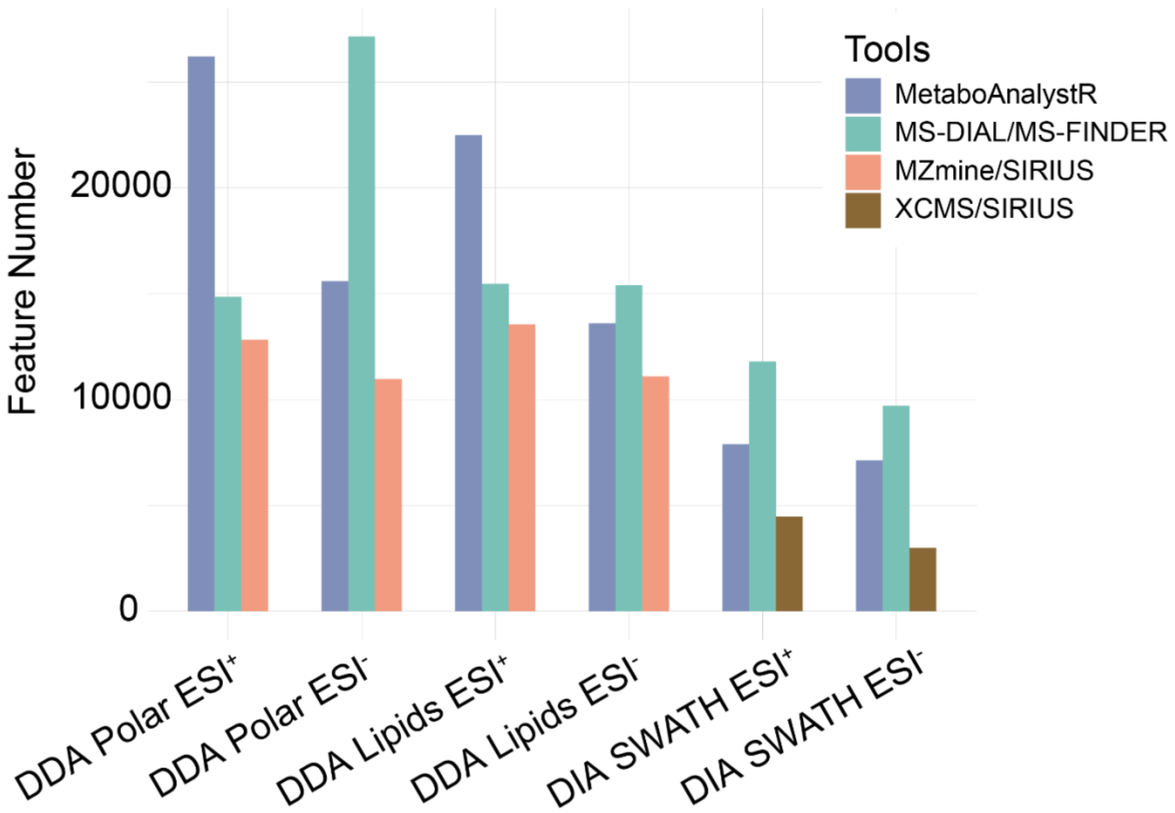

Supplementary Fig. 18. Graphical summary of MS1 features detected by different tools from clinical metabolomics datasets. Summary of MS1 features detected by different tools from DDA and SWATH-DIA datasets.

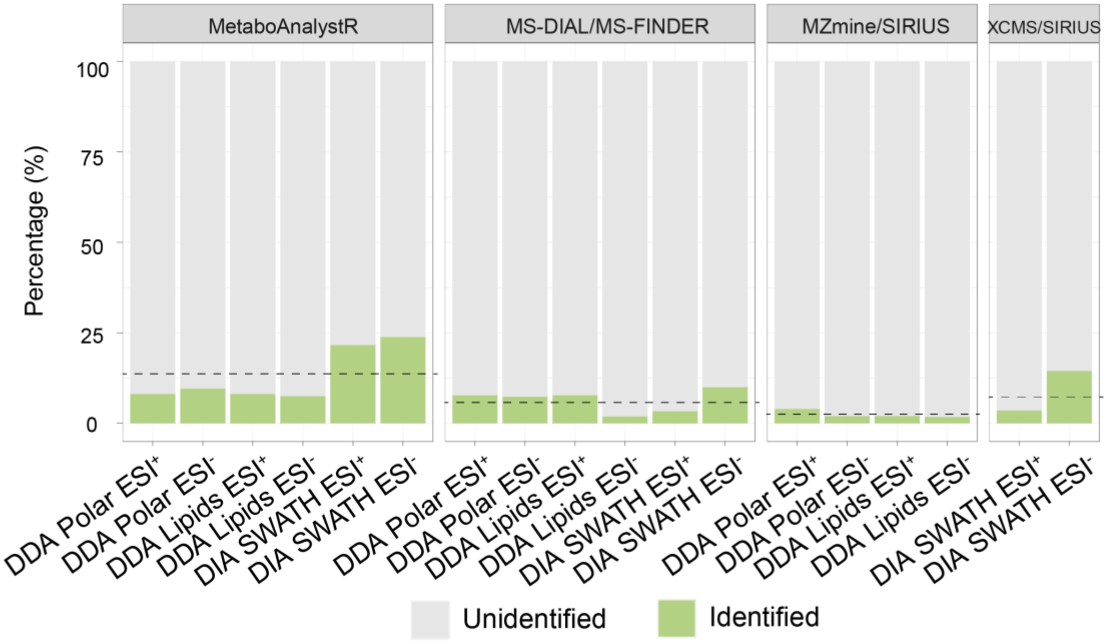

Supplementary Fig. 19. Percentage of MS1 features identified with MS2 spectra from clinical metabolomics datasets. The dotted line indicates the average percentage of the compound identification.

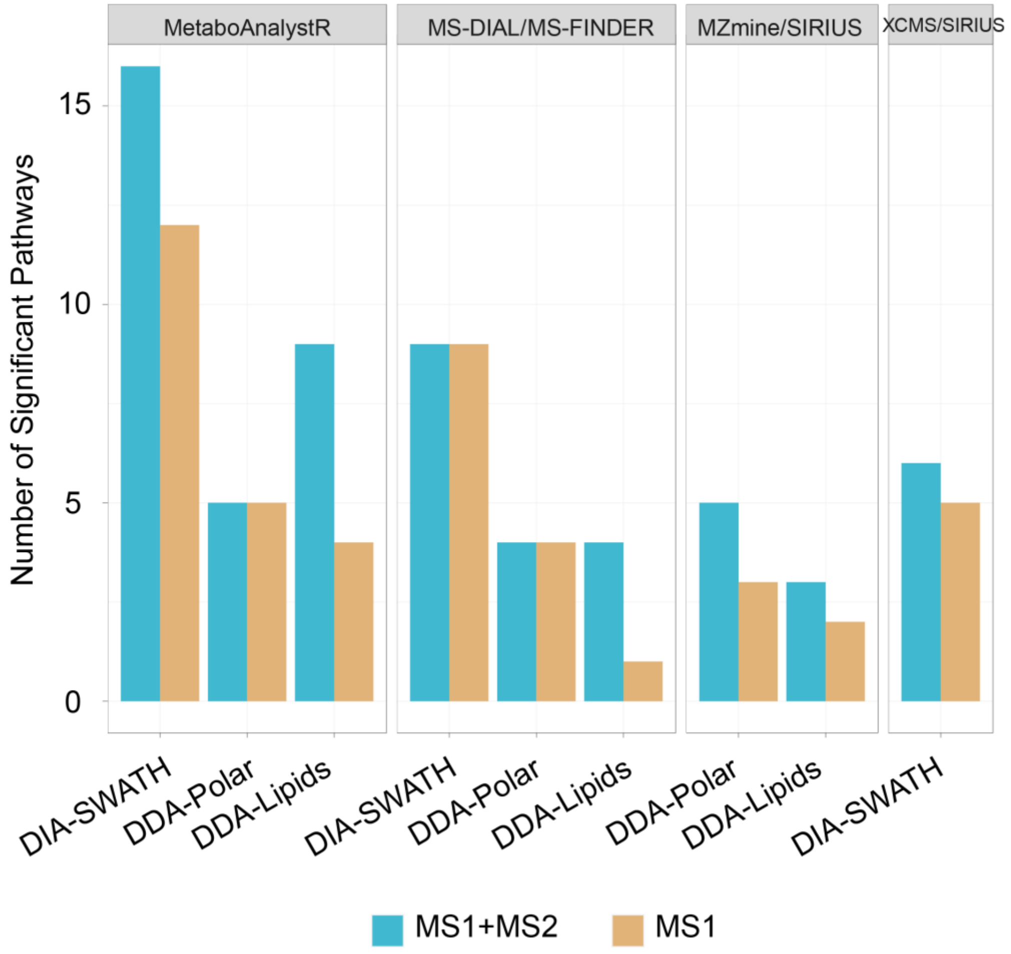

Supplementary Fig. 20. Summary of pathway analysis results from different tools as well as the effects of integrating MS2 identification results (MS1+MS2) or not (MS1).

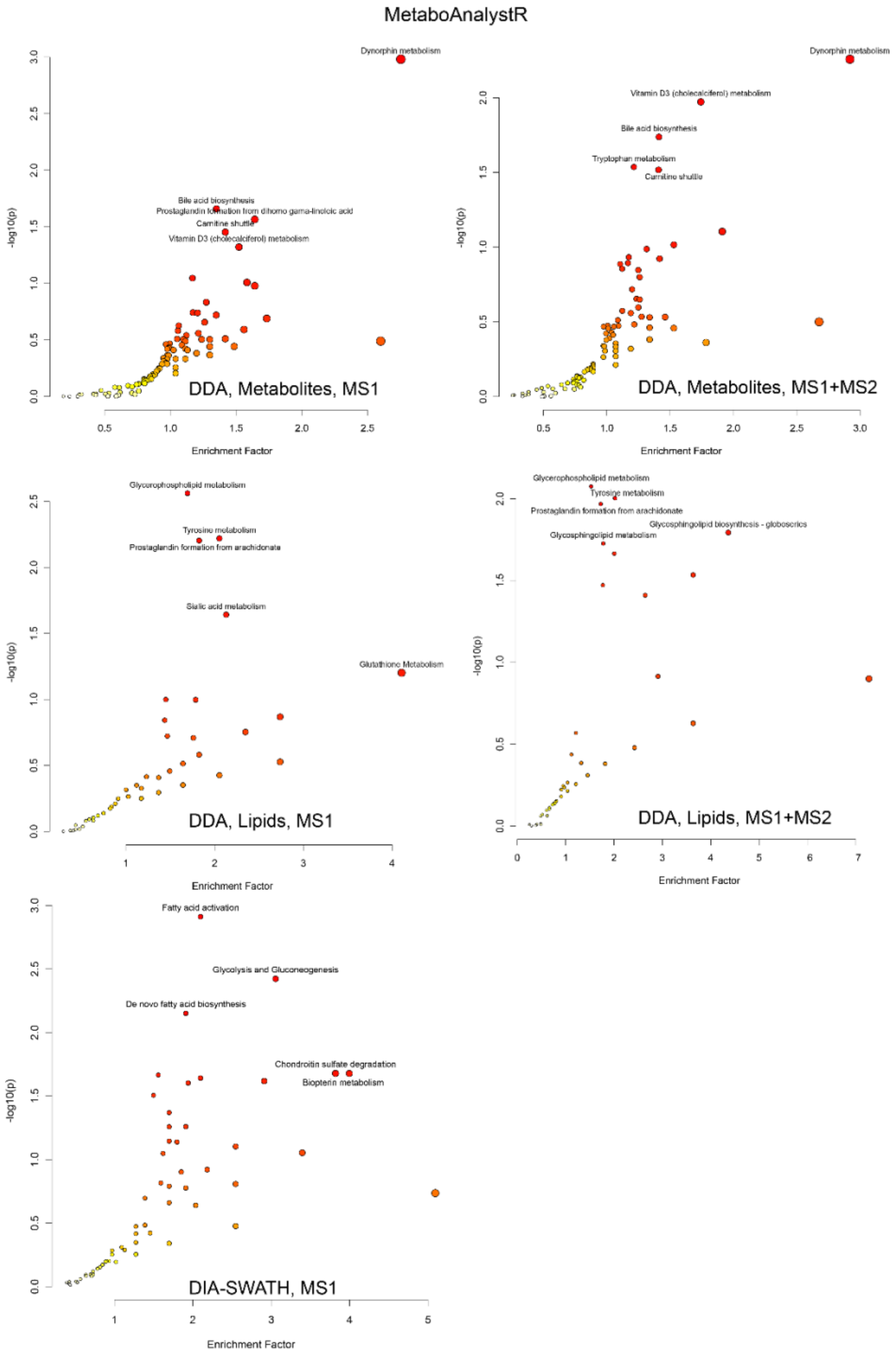

Supplementary Fig. 21. Scatter plots of pathway analysis results based on MS1 features and MS2 compounds from MetaboAnalystR. In most cases, integrating MS2 results into the pipeline increases the discovery of pathways or improves the statistical significance compared to using MS1 alone. n=10 independent experiment samples of two groups for SWATH-DIA dataset. n=16

independent experiment samples for Mild group and n=54 independent experiment samples for fatal group for DDA dataset. Fisher's exact test without adjustment for functional analysis.

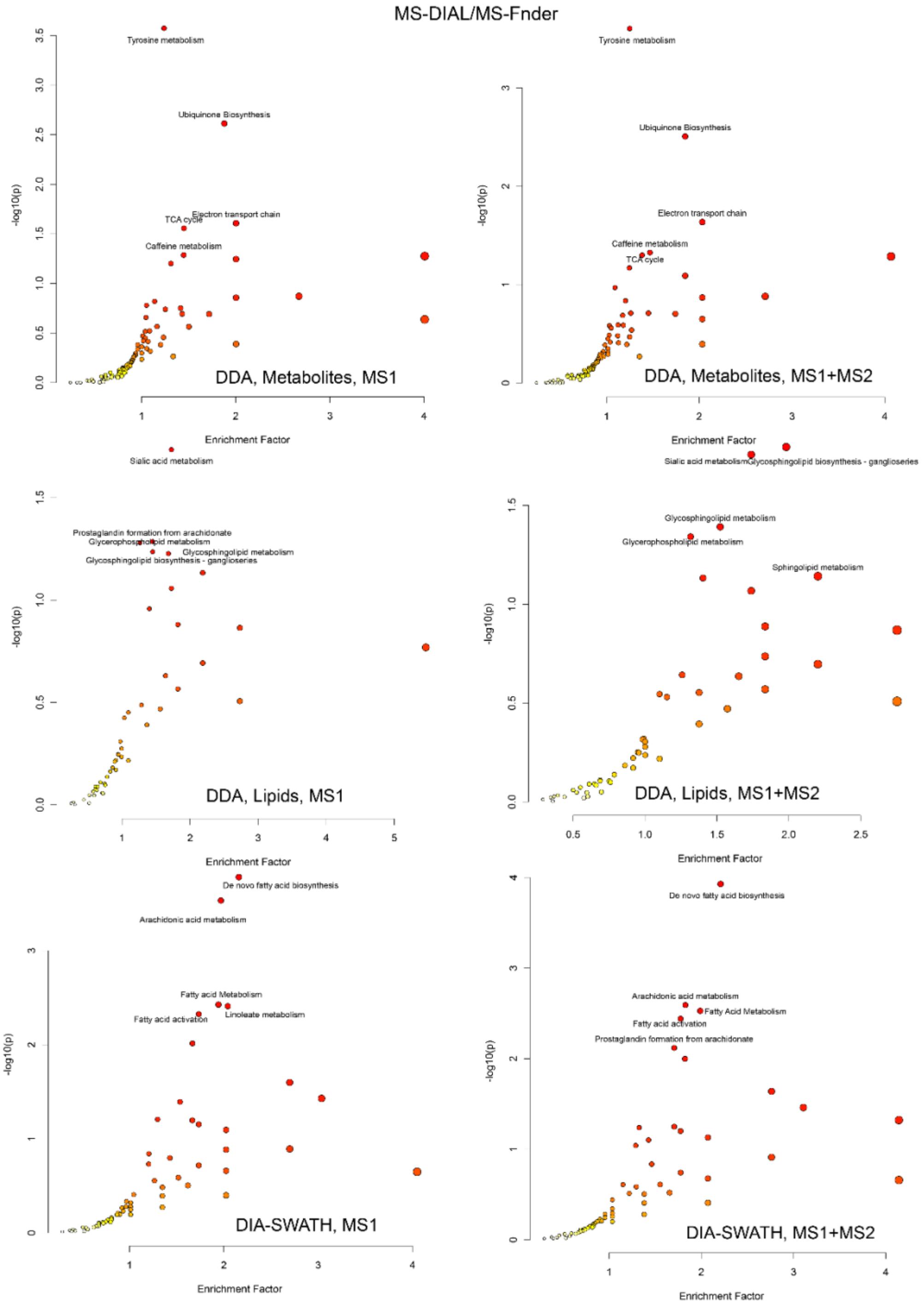

Supplementary Fig. 22. Scatter plots of pathway analysis results based on MS1 features and MS2 compounds using MS-DIAL/MS-Finder. Pathway analysis was performed by MetaboAnalystR. In most cases, integrating MS2 results into the pipeline increases the discovery of pathways or improves the statistical significance compared to using MS1 alone. n=10 independent experiment

samples of two groups for SWATH-DIA dataset. n=16 independent experiment samples for Mild group and n=54 independent experiment samples for fatal group for DDA dataset. Fisher's exact test without adjustment for functional analysis.

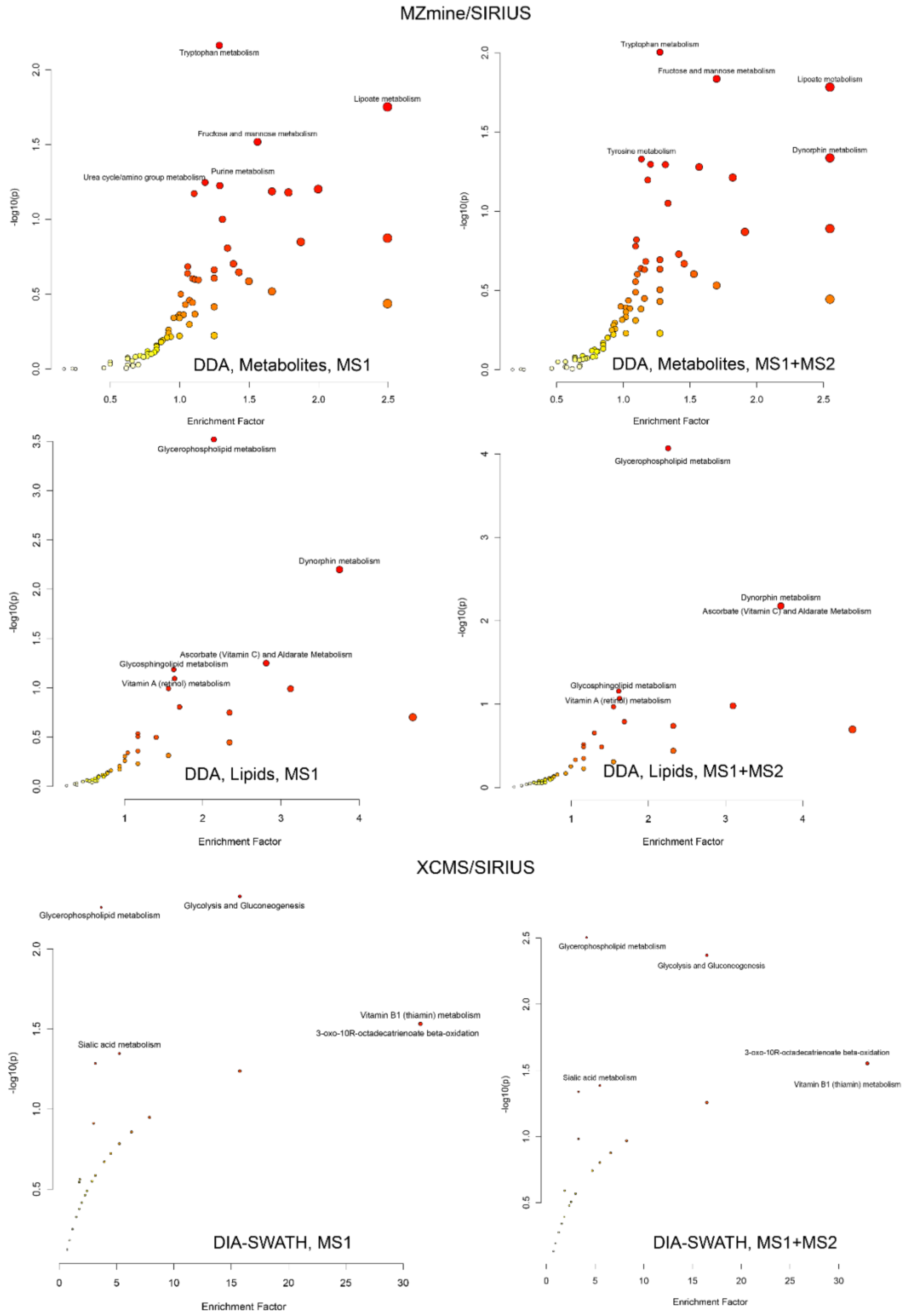

Supplementary Fig. 23. Scatter plots of pathway analysis results based on MS1 features and MS2 compounds from MZmine/SIRIUS (for DDA) or XCMS/SIRIUS (for SWATH-DIA). Pathway

analysis was performed by MetaboAnalystR. In most cases, integrating MS2 results into the pipeline increases the discovery of pathways or improves the statistical significance compared to using MS1 alone. n=10 independent experiment samples of two groups for SWATH-DIA dataset. n=16 independent experiment samples for Mild group and n=54 independent experiment samples for fatal group for DDA dataset. Fisher's exact test without adjustment for functional analysis.

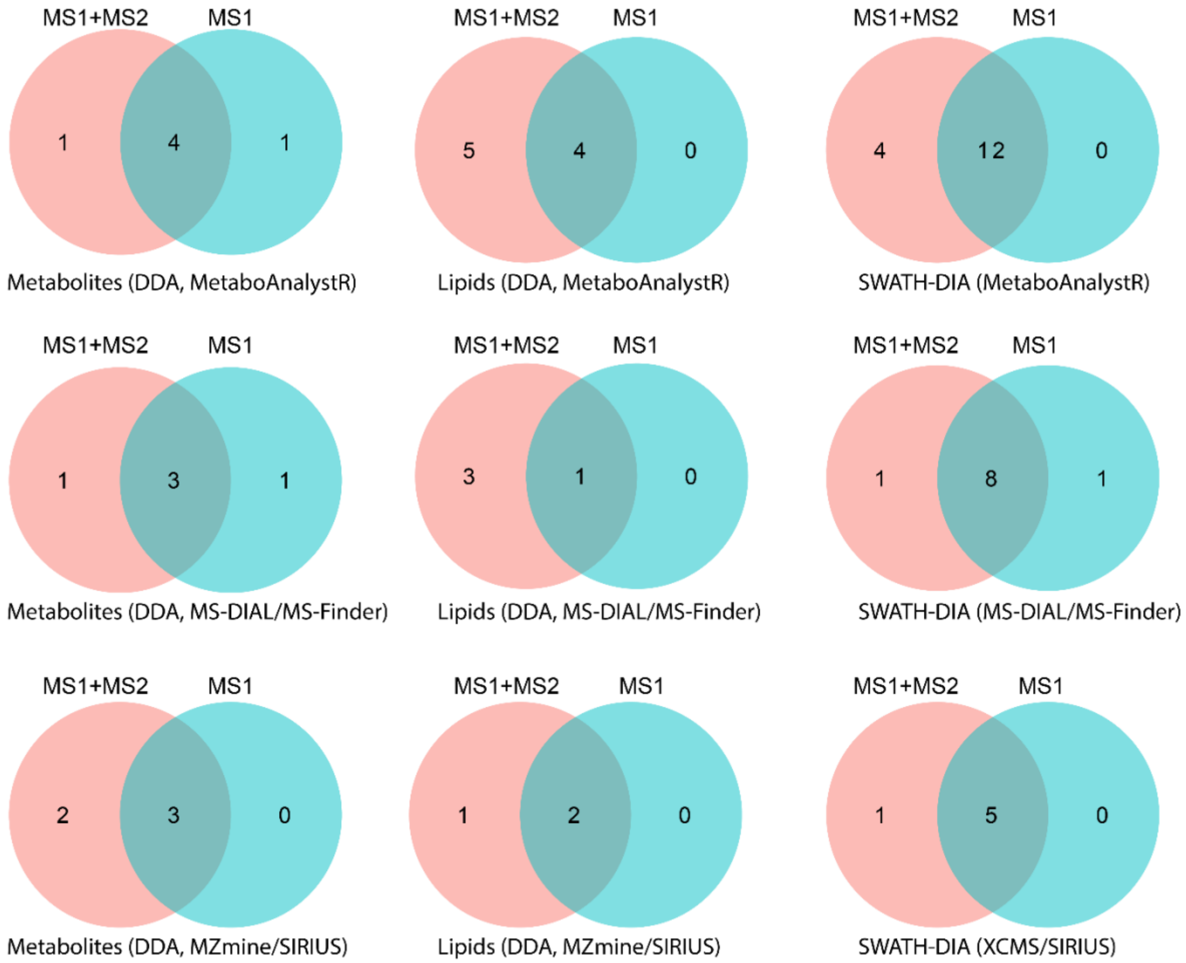

Supplementary Fig. 24. Venn diagram of pathway analysis results from different datasets. These diagrams summarize the intersections of pathways identified by MetaboAnalystR *Mummichog* function on the MS1 features or MS2 features from different tools. In most cases, integrating MS2 results in *Mummichog* functions increases the discovery of pathways or improves the statistical significance compared to using MS1 alone.

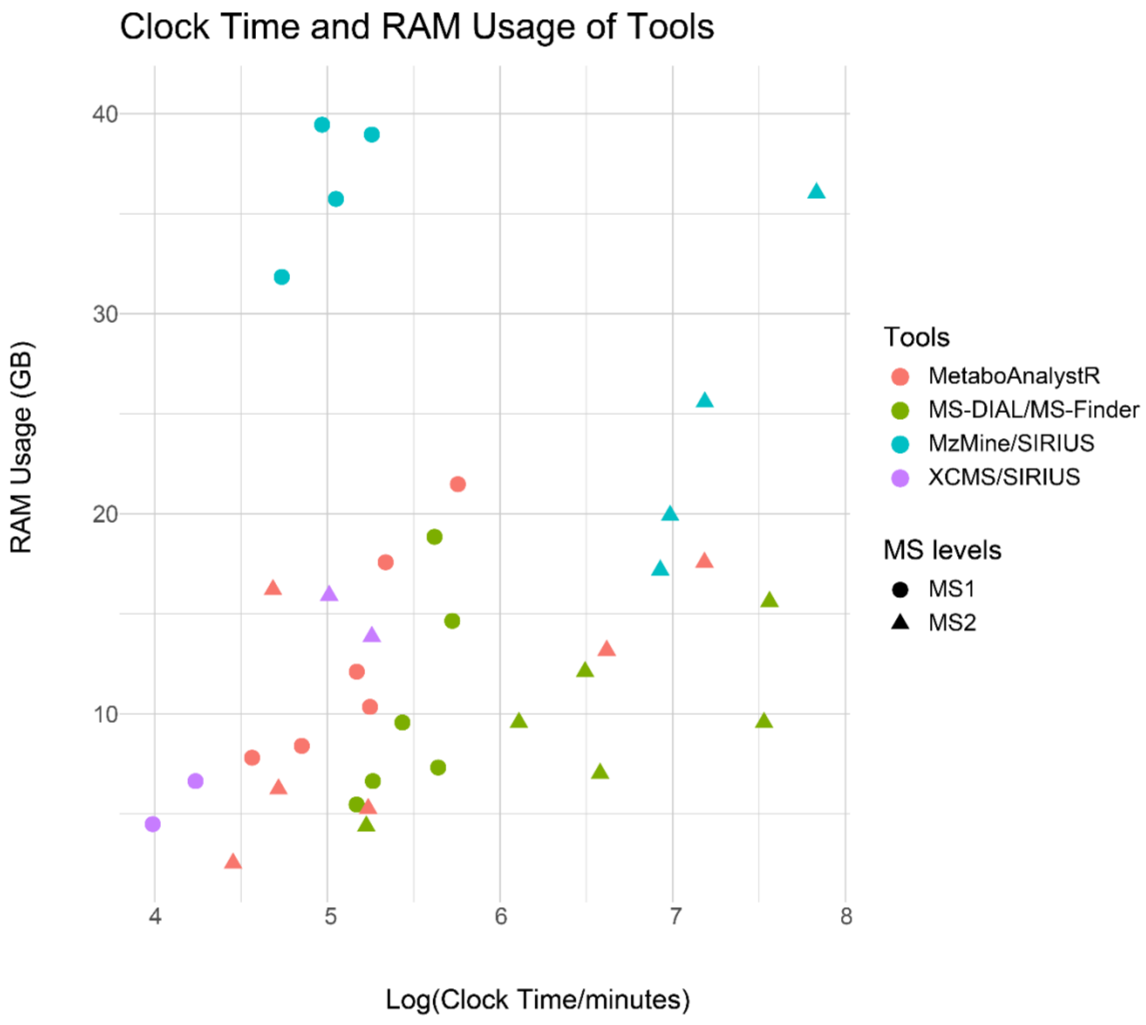

Supplementary Fig. 25. Comparison of memory usage and computing time of different tools. Overall, MetaboAnalystR used less RAM and finished both MS1 and MS2 detection in an efficient way. MS-DIAL/MS-Finder usually consumed less RAM, but more time. MZmine could finish the MS1 features detection in less time, but with higher memory consumption. SIRIUS could be very slow due to the remote API access.

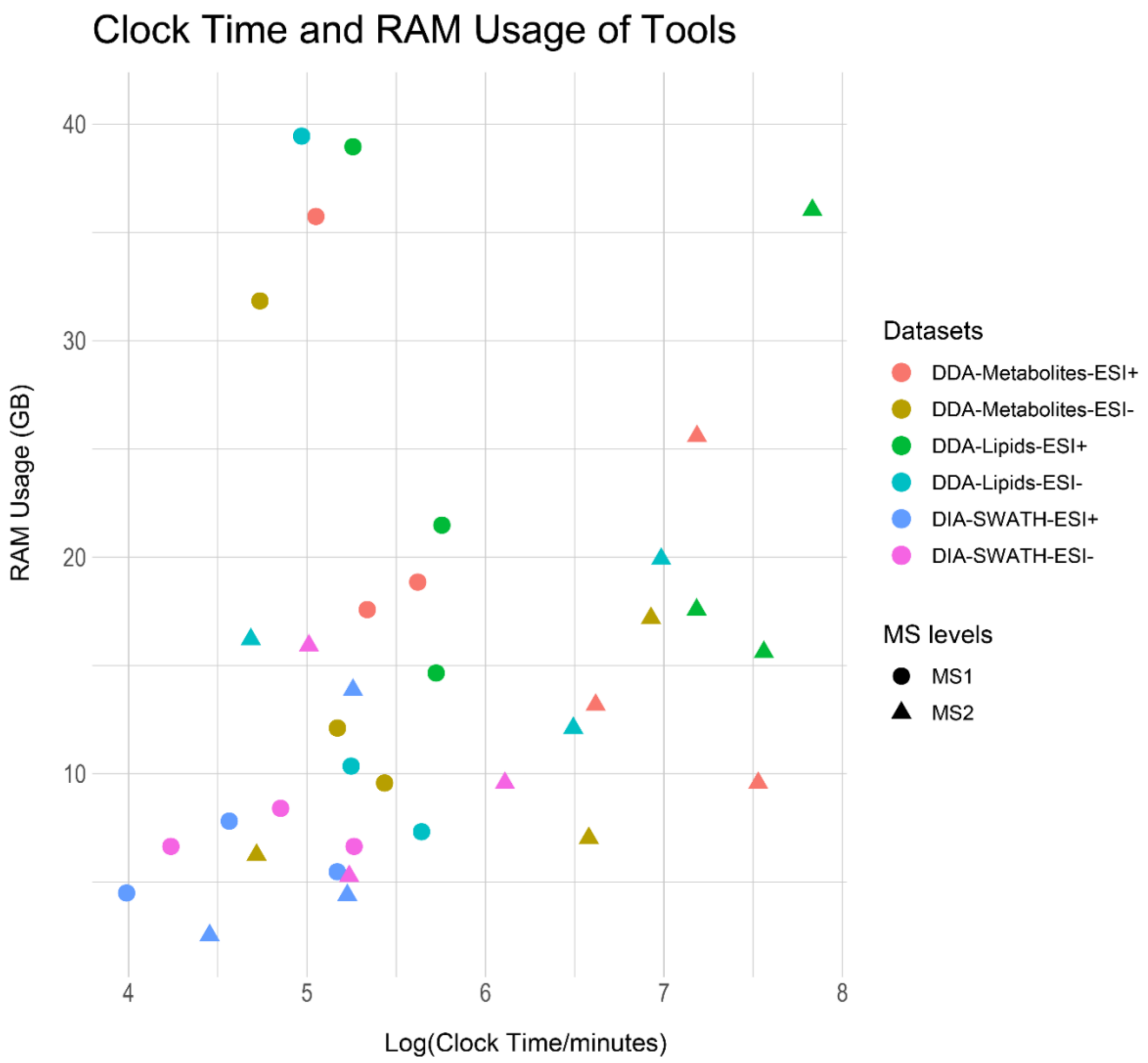

Supplementary Fig. 26. Summary of computational efficiencies of all tools for different datasets. This figure accompanies supplementary Fig. 25 above. It displays efficiency scores in different colors based on datasets. Processing of SWATH-DIA datasets by various tools is notably faster than other datasets, which is attributed to the smaller size of SWATH-DIA (n=30) compared to others (n=160). The computational performance shows no clear difference among DDA datasets, suggesting that the computational efficiency is primarily determined by the tools used, rather than by the datasets.

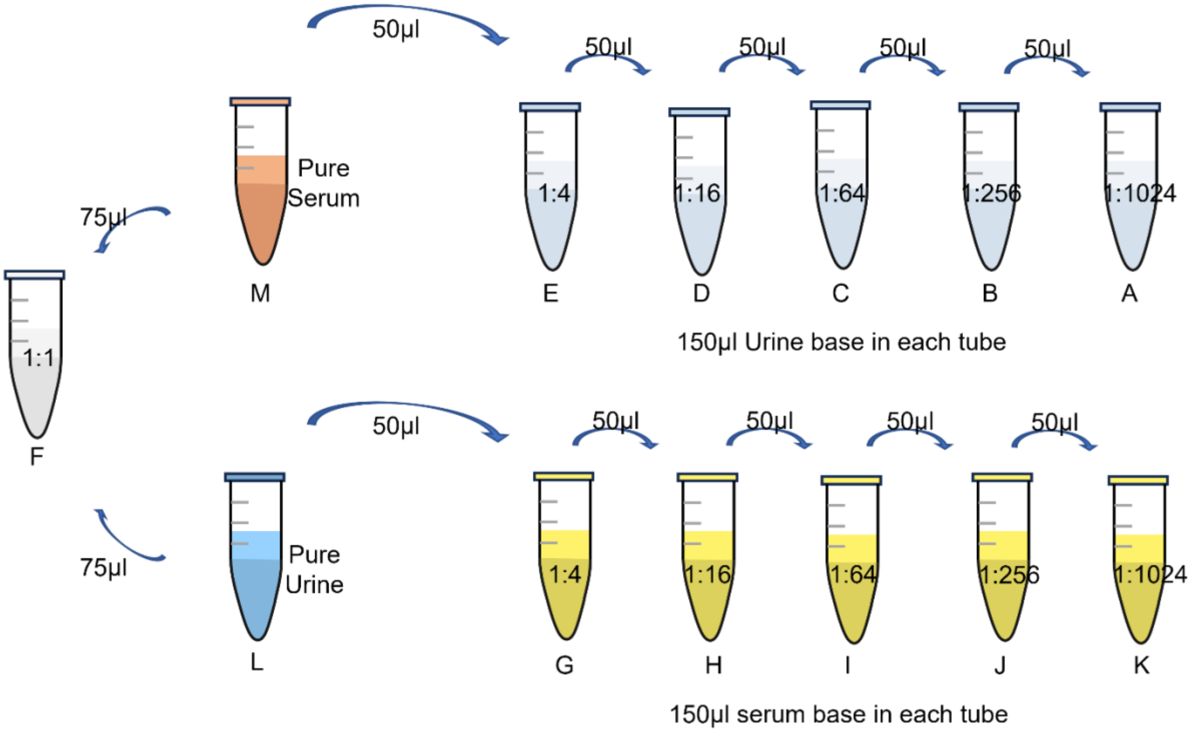

Supplementary Fig. 27. Graphical illustration of preparation of serial dilution samples. To begin, tubes A to E are filled with 150 μl of base urine, while tubes G to K are filled with 150 μl of base serum. The quadruple dilution process is carried out by adding urine to serum or serum to urine in a quadruple dilution manner. Tube F contains an equal volume of serum and urine.

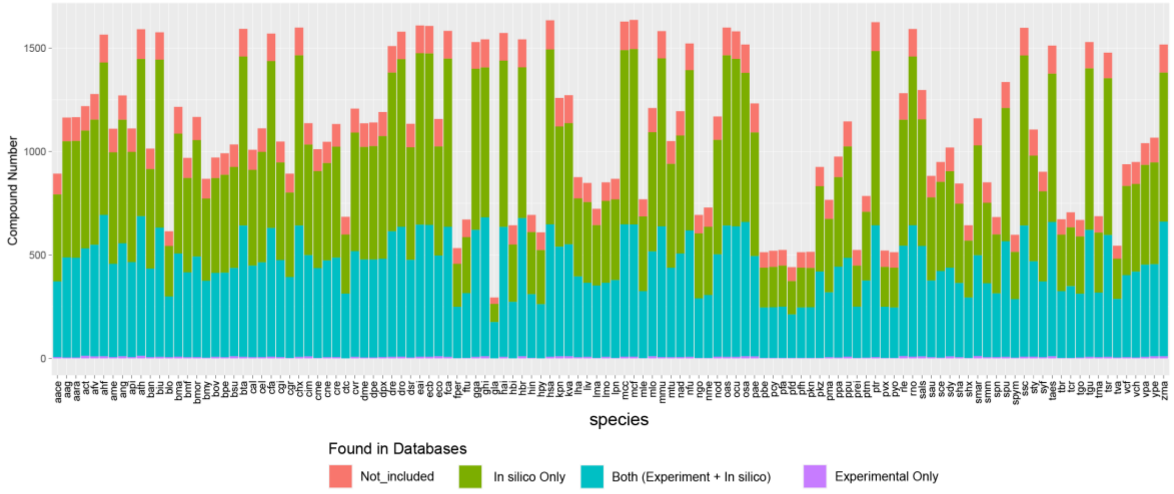

Supplementary Fig. 28. Graphical summary of compounds in the pathway reference library across 120 species. The summary encompasses all 3,456 compounds in the database, with > 90% of the compounds having MS2 records included, and > 50% of the compounds in the library feature both experimental and *in-silico* MS2 spectra.

ID	CompoundName	DBID	PrecursorMZ	PrecursorType	Formula	Smiles	InchiKey	InstrumentType	CollisionEnergy	RetentionTime	Ontology	NumberOfPeak	MS2Peaks
Filter	Filter	Filter	Filter	Filter	Filter	Filter	Filter	Filter	Filter	Filter	Filter	Filter	Filter
1	Pyruvic acid	BMDM...	89.02	[M+H]+	C3H4O3	O=C(O)C...	LCTONWC...	Orbitrap	10.0	2.2	Alpha-keto...	7	68.952 2...
2	Pyruvic acid	BMDM...	111.01	[M+Na]+	C3H4O3	O=C(O)C...	LCTONWC...	Orbitrap	10.0	2.2	Alpha-keto...	4	110.975 69...
3	Pyruvic acid	BMDM...	89.02	[M+H]+	C3H4O3	O=C(O)C...	LCTONWC...	Orbitrap	10.0	2.2	Alpha-keto...	18	68.452 21...
4	Pyruvic acid	BMDM...	111.01	[M+Na]+	C3H4O3	O=C(O)C...	LCTONWC...	Orbitrap	10.0	2.2	Alpha-keto...	4	110.975 64...
5	Pyruvic acid	BMDM...	89.02	[M+H]+	C3H4O3	O=C(O)C...	LCTONWC...	Orbitrap	10.0	2.2	Alpha-keto...	21	67.951 7...
6	Pyruvic acid	BMDM...	111.01	[M+Na]+	C3H4O3	O=C(O)C...	LCTONWC...	Orbitrap	10.0	2.2	Alpha-keto...	5	110.975 76...
7	Pyruvic acid	BMDM...	89.02	[M+H]+	C3H4O3	O=C(O)C...	LCTONWC...	Orbitrap	10.0	2.2	Alpha-keto...	20	68.452 14...
8	Pyruvic acid	BMDM...	111.01	[M+Na]+	C3H4O3	O=C(O)C...	LCTONWC...	Orbitrap	10.0	2.2	Alpha-keto...	7	83.049 10...
9	Pyruvic acid	BMDM...	89.02	[M+H]+	C3H4O3	O=C(O)C...	LCTONWC...	Orbitrap	10.0	2.2	Alpha-keto...	19	68.452 77...
10	Pyruvic acid	BMDM...	111.01	[M+Na]+	C3H4O3	O=C(O)C...	LCTONWC...	Orbitrap	10.0	2.2	Alpha-keto...	8	83.049 15...
11	Pyruvic acid	BMDM...	89.02	[M+H]+	C3H4O3	O=C(O)C...	LCTONWC...	Orbitrap	10.0	2.2	Alpha-keto...	19	68.452 28...
12	Pyruvic acid	BMDM...	111.01	[M+Na]+	C3H4O3	O=C(O)C...	LCTONWC...	Orbitrap	10.0	2.2	Alpha-keto...	6	110.975 94...
13	Pyruvic acid	BMDM...	89.02	[M+H]+	C3H4O3	O=C(O)C...	LCTONWC...	Orbitrap	10.0	2.2	Alpha-keto...	21	67.951 12...
14	Pyruvic acid	BMDM...	111.01	[M+Na]+	C3H4O3	O=C(O)C...	LCTONWC...	Orbitrap	10.0	2.2	Alpha-keto...	4	110.975 74...
15	Pyruvic acid	BMDM...	89.02	[M+H]+	C3H4O3	O=C(O)C...	LCTONWC...	Orbitrap	10.0	2.2	Alpha-keto...	20	67.951 10...
16	Pyruvic acid	BMDM...	111.01	[M+Na]+	C3H4O3	O=C(O)C...	LCTONWC...	Orbitrap	10.0	2.2	Alpha-keto...	5	110.975 31...
17	Pyruvic acid	BMDM...	89.02	[M+H]+	C3H4O3	O=C(O)C...	LCTONWC...	Orbitrap	10.0	2.2	Alpha-keto...	18	67.951 6...
18	Pyruvic acid	BMDM...	111.01	[M+Na]+	C3H4O3	O=C(O)C...	LCTONWC...	Orbitrap	10.0	2.2	Alpha-keto...	7	110.975 29...
19	Pyruvic acid	BMDM...	89.02	[M+H]+	C3H4O3	O=C(O)C...	LCTONWC...	Orbitrap	10.0	2.2	Alpha-keto...	20	68.452 25...
20	Pyruvic acid	BMDM...	111.01	[M+Na]+	C3H4O3	O=C(O)C...	LCTONWC...	Orbitrap	10.0	2.2	Alpha-keto...	6	110.975 65...
21	Pyruvic acid	BMDM...	89.02	[M+H]+	C3H4O3	O=C(O)C...	LCTONWC...	Orbitrap	10.0	2.2	Alpha-keto...	22	68.452 48...
22	Pyruvic acid	BMDM...	111.01	[M+Na]+	C3H4O3	O=C(O)C...	LCTONWC...	Orbitrap	10.0	2.2	Alpha-keto...	3	110.975 88...

Supplementary Fig. 29. The structure of MS2 reference library required by MetaboAnalystR. The reference library is based on an SQLite database, which supports multiple tables. Each table in the database should contain the following mandatory columns: ID, CompoundName, PrecursorMZ, PrecursorType, Formula, InChIKeys, and MS2Peaks. Other columns are not mandatory.

Supplementary Table 1. Summary of identified compounds by different tools (DDA, ESI^-). MS-DIAL, MS-FINDER, MZmine and SIRIUS were evaluated under their default parameter settings.

Tools	Number of detected standards (MS1) *	Compounds correctly annotated (MS2)
MS-DIAL + MS-FINDER	271	121 (26.4%)
MZmine + SIRIUS	317	124 (27.0%)
MetaboAnalystR	336	194 (42.3%)
MetaboAnalystR (nonDeco)	336	185 (40.3%)

* This standard mixture contains a total of 459 detectable compounds under ESI^-.

Supplementary Table 2. Summary of identified compounds by different tools (SWATH-DIA ESI^+). MS-DIAL, MS-FINDER, MZmine and SIRIUS were evaluated under their default parameter settings.

Tools	Number of detected standards (MS1) *	Compounds correctly annotated (MS2)
MS-DIAL + MS-FINDER	5	1 (0.25%)
XCMS + SIRIUS	108	42 (10.3%)
MetaboAnalystR	324	143 (35.22%)
MetaboAnalystR (PathwayDB)	324	148 (36.45%)

* This standard mixture contains a total of 406 detectable compounds under ESI^+.

Supplementary Table 3. Summary of identified compounds by different tools (SWATH-DIA ESI^-). MS-DIAL, MS-FINDER, MZmine and SIRIUS were evaluated under their default parameter settings.

Tools	Number of detected standards (MS1) *	Compounds correctly annotated (MS2)
MS-DIAL + MS-FINDER	6	3 (0.65%)
XCMS + SIRIUS	107	46 (10.02%)
MetaboAnalyst	241	102 (22.22%)
MetaboAnalyst (PathwayDB)	241	97 (21.13%)

* This standard mixture contains a total of 459 detectable compounds under ESI^-.

Supplementary Table 4. Pathway enrichment results of polar metabolites datasets from three tools

Pathways	Pathway total	Hits.total	Hits.sig	Expected	P value	Results	Tools
Fatty acid activation	74	35	14	6.6766	0.0012	MS1	MetaboAnalystR
Glycolysis and Gluconeogenesis	49	16	13	1.9637	0.0037	MS1	
De novo fatty acid biosynthesis	106	30	10	6.2838	0.0070	MS1	
Chondroitin sulfate degradation	37	4	4	0.78548	0.0209	MS1	
Biopterin metabolism	22	3	3	0.78548	0.0209	MS1	
Heparan sulfate degradation	34	4	4	0.78548	0.0209	MS1	
Glycerophospholipid metabolism	156	35	14	9.6221	0.0216	MS1	
Butanoate metabolism	34	14	8	3.3383	0.0228	MS1	
TCA cycle	31	8	4	1.3746	0.0241	MS1	
Arachidonic acid metabolism	95	30	23	4.1238	0.0249	MS1	
Tyrosine metabolism	160	51	18	10.015	0.0311	MS1	
Leukotriene metabolism	92	28	13	5.302	0.0427	MS1	
Fatty acid activation	74	27	12	5.8327	0.0009	MS1+MS2	
De novo fatty acid biosynthesis	106	26	10	5.8327	0.0035	MS1+MS2	
Leukotriene metabolism	92	21	12	3.8214	0.0039	MS1+MS2	
Tyrosine metabolism	160	44	18	9.0508	0.0123	MS1+MS2	
Arachidonic acid metabolism	95	18	13	3.8214	0.0154	MS1+MS2	
Prostaglandin formation from arachidonate	78	19	8	3.218	0.0186	MS1+MS2	
Glycerophospholipid metabolism	156	32	13	9.453	0.0189	MS1+MS2	
Phosphatidylinositol phosphate metabolism	59	16	9	2.6147	0.0221	MS1+MS2	
Chondroitin sulfate degradation	37	3	3	0.80451	0.0229	MS1+MS2	
Biopterin metabolism	22	3	3	0.80451	0.0229	MS1+MS2	
Heparan sulfate degradation	34	3	3	0.80451	0.0229	MS1+MS2	
Glycolysis and Gluconeogenesis	49	13	11	1.4079	0.0269	MS1+MS2	
Butanoate metabolism	34	10	6	2.8158	0.0328	MS1+MS2	
Vitamin D3 (cholecalciferol) metabolism	16	10	5	2.2124	0.0394	MS1+MS2	
Ascorbate (Vitamin C) and Aldarate Metabolism	29	13	7	3.0169	0.0463	MS1+MS2	
TCA cycle	31	6	3	1.0056	0.0495	MS1+MS2	
De novo fatty acid biosynthesis	106	32	15	7.4104	0.0001	MS1	MS-DIAL MS-FINDER
Arachidonic acid metabolism	95	54	45	9.1394	0.0002	MS1	
Linoleate metabolism	46	23	13	6.1753	0.0037	MS1	
Fatty Acid Metabolism	63	18	9	6.1753	0.0037	MS1	
Fatty acid activation	74	36	14	8.6454	0.0047	MS1	
Prostaglandin formation from arachidonate	78	40	18	8.3984	0.0096	MS1	
Heparan sulfate degradation	34	6	5	1.4821	0.025	MS1	

Chondroitin sulfate degradation	37	4	4	0.98805	0.0372	MS1	
Glycosphingolipid metabolism	67	23	15	7.1633	0.0404	MS1	
De novo fatty acid biosynthesis	106	32	15	7.2442	0.0001	MS1+MS2	
Arachidonic acid metabolism	95	53	42	8.2101	0.0025	MS1+MS2	
Fatty Acid Metabolism	63	18	9	6.0368	0.0029	MS1+MS2	
Fatty acid activation	74	36	14	8.4516	0.0036	MS1+MS2	
Prostaglandin formation from arachidonate	78	39	17	8.2101	0.0076	MS1+MS2	
Linoleate metabolism	46	23	13	6.0368	0.0100	MS1+MS2	
Heparan sulfate degradation	34	6	5	1.4488	0.0229	MS1+MS2	
Chondroitin sulfate degradation	37	4	4	0.96589	0.0346	MS1+MS2	
Prostaglandin formation from dihomo gama-linoleic acid	11	1	1	0.48295	0.0476	MS1+MS2	
Glycolysis and Gluconeogenesis	49	5	4	0.12698	0.0046	MS1	XCMS SIRIUS
Glycerophospholipid metabolism	156	30	6	1.3651	0.0055	MS1	
3-oxo-10R-octadecatrienoate beta-oxidation	27	4	4	0.031746	0.0293	MS1	
Vitamin B1 (thiamin) metabolism	20	1	1	0.031746	0.0293	MS1	
Sialic acid metabolism	107	14	6	0.38095	0.0449	MS1	
Glycerophospholipid metabolism	156	28	6	1.2146	0.0031	MS1+MS2	
Glycolysis and Gluconeogenesis	49	5	4	0.12146	0.0042	MS1+MS2	
3-oxo-10R-octadecatrienoate beta-oxidation	27	4	4	0.030364	0.0280	MS1+MS2	
Vitamin B1 (thiamin) metabolism	20	1	1	0.030364	0.0280	MS1+MS2	
Sialic acid metabolism	107	13	6	0.36437	0.0412	MS1+MS2	
Glycosphingolipid metabolism	67	21	7	0.91093	0.0458	MS1+MS2	

Fisher's exact test without adjustment for functional analysis

Supplementary Table 5. Pathway enrichment results of polar metabolites datasets from three tools

Pathways	Pathway total	Hits.total	Hits.sig	Expected	P value	Methods	Tools
Dynorphin metabolism	8	5	4	3.2683	0.0010	MS1	MetaboAnalystR
Bile acid biosynthesis	82	56	45	19.994	0.0219	MS1	
Prostaglandin formation from dihomo gama-linoleic acid	11	8	6	7.3056	0.0271	MS1	
Carnitine shuttle	72	30	18	12.689	0.0352	MS1	
Vitamin D3 (cholecalciferol) metabolism	16	14	12	7.8823	0.0477	MS1	
Dynorphin metabolism	8	5	3	2.0546	0.00552	MS1+MS2	
Vitamin D3 (cholecalciferol) metabolism	16	14	13	8.0316	0.0106	MS1+MS2	
Bile acid biosynthesis	82	55	39	16.997	0.01829	MS1+MS2	
Tryptophan metabolism	94	65	36	36.236	0.0290	MS1+MS2	
Carnitine shuttle	72	32	21	14.195	0.0303	MS1+MS2	
Tyrosine metabolism	160	100	70	82.367	0.0002	MS1	MS-DIAL MS-FINDER
Ubiquinone Biosynthesis	10	8	6	7.9871	0.00244	MS1	
Electron transport chain	7	2	2	3.4944	0.0246	MS1	
TCA cycle	31	17	11	11.731	0.0277	MS1	
Tyrosine metabolism	160	99	68	79.193	0.0002	MS1+MS2	
Ubiquinone Biosynthesis	10	8	6	8.1161	0.0031	MS1+MS2	
Electron transport chain	7	2	2	3.4432	0.0230	MS1+MS2	
Caffeine metabolism	11	10	8	8.8539	0.0471	MS1+MS2	
Tryptophan metabolism	94	69	41	27.25	0.0068	MS1	MZmine SIRIUS
Lipoate metabolism	8	4	4	1.6029	0.0176	MS1	
Fructose and mannose metabolism	33	27	22	6.4118	0.0303	MS1	
Tryptophan metabolism	94	69	40	26.683	0.0099	MS1+MS2	
Fructose and mannose metabolism	33	25	21	5.8859	0.0146	MS1+MS2	
Lipoate metabolism	8	4	4	1.5696	0.0164	MS1+MS2	
Dynorphin metabolism	8	3	3	1.1772	0.04609	MS1+MS2	
Tyrosine metabolism	160	103	54	36.1	0.046879	MS1+MS2	

Fisher's exact test without adjustment for functional analysis

Supplementary Table 6. Pathway enrichment results of lipids datasets from three tools

Pathways	Pathway total	Hits.total	Hits.sig	Expected	P value	Methods	Tools
Glycerophospholipid metabolism	156	31	13	11.809	0.00274	MS1	MetaboAnalystR
Tyrosine metabolism	160	35	10	5.3565	0.00603	MS1	
Prostaglandin formation from arachidonate	78	57	11	7.6696	0.00625	MS1	
Sialic acid metabolism	107	18	4	3.287	0.02279	MS1	
Glycerophospholipid metabolism	156	31	14	13.746	0.00841	MS1+MS2	
Tyrosine metabolism	160	25	10	4.9485	0.00993	MS1+MS2	
Prostaglandin formation from arachidonate	78	53	13	8.11	0.01077	MS1+MS2	
Glycosphingolipid biosynthesis - globoseries	16	4	2	0.68729	0.01609	MS1+MS2	
Glycosphingolipid metabolism	67	23	7	6.1856	0.01877	MS1+MS2	
Glycosphingolipid biosynthesis - ganglioseries	62	10	6	3.9863	0.02166	MS1+MS2	
Fructose and mannose metabolism	33	12	4	0.82474	0.02925	MS1+MS2	
Sialic acid metabolism	107	18	5	5.0859	0.03374	MS1+MS2	
Sphingolipid metabolism	3	3	2	1.512	0.03896	MS1+MS2	
Sialic acid metabolism	107	26	8	6.9532	0.01843	MS1	MS-DIAL MS-FINDER
Glycosphingolipid biosynthesis - ganglioseries	62	13	6	4.5404	0.01600	MS1+MS2	
Sialic acid metabolism	107	26	8	6.9014	0.01749	MS1+MS2	
Glycosphingolipid metabolism	67	24	11	8.536	0.04060	MS1+MS2	
Glycerophospholipid metabolism	156	33	16	16.709	0.04551	MS1+MS2	
Glycerophospholipid metabolism	156	32	15	7.4704	0.00030	MS1	MZmine SIRIUS
Dynorphin metabolism	8	5	4	1.0672	0.00632	MS1	
Glycerophospholipid metabolism	156	32	16	7.5352	8.54E-05	MS1+MS2	
Ascorbate (Vitamin C) and Aldarate Metabolism	29	5	4	1.0765	0.00664	MS1+MS2	
Dynorphin metabolism	8	5	4	1.0765	0.00664	MS1+MS2	

Fisher's exact test without adjustment for functional analysis

Supplementary Table 7. Comparison of computational performance of different tools. The values are presented as relative values to the MS-DIAL/MS-FINDER workflow.

Modes	Comparisons	Tools	MS1		MS2		Mean
			DDA (Polar*)	DDA (Lipids)	DDA (Polar)	DDA (Lipids)	
DDA	Clock Time Elapse	MS-DIAL/MS-FINDER	1	1	1	1	1
		MetaboAnalystR	0.76	0.86	0.33	0.55	0.62
		MZmine/SIRIUS	0.53	0.57	0.91	1.39	0.85
	RAM Usage	MS-DIAL/MS-FINDER	1	1	1	1	1
		MetaboAnalystR	1.04	1.45	1.17	1.22	1.22
		MZmine/SIRIUS	2.38	3.57	2.57	2.02	2.64
SWATH-DIA	Clock Time Elapse	MS-DIAL/MS-FINDER	1		1		1
		MetaboAnalystR	0.61		0.43		0.52
		XCMS/SIRIUS	0.33		0.54		0.44
	RAM Usage	MS-DIAL/MS-FINDER	1		1		1
		MetaboAnalystR	1.34		0.56		0.95
		XCMS/SIRIUS	0.91		2.13		1.52

* Polar, polar compounds

Supplementary Table 8. Demographics of all subjects involved in current study

Items	Values
Age	28.4 ± 5.6
Gender (F/M)	12 (5/7)
BMI	22.7 ± 4.1
Ethnicities	Caucasian/South Asian/East Asian/Latino/Mixed (7/1/2/1/1)
Diabetes	None
Pregnant	None
Breastfeeding	None

Supplementary Table 9. Chromatographic conditions, gradient procedure instrumental settings

Columns	Time (min)	Flow rate (mL/min)	A (%)	B (%)
C18	0	0.4	95	5
	1	0.4	95	5
	3	0.4	50	50
	15	0.4	20	80
	15.5	0.4	0	100
	19.5	0.4	0	100
	20	0.4	95	5
	Column Temperature	40℃		
	Autosampler Temperature	4℃		
	Injection Volume	5μL		
HILIC	Time (min)	Flow rate (mL/min)	A (%)	B (%)
	0	0.4	1	99
	3	0.4	1	99
	20	0.4	50	50
	21	0.4	95	5
	24	0.4	95	5
	25	0.4	1	99
	35	STOP		
	Column Temperature	35℃		
	Autosampler Temperature	4℃		
	Injection Volume	1μL		

Supplementary Table 10. Parameters of mass spectrometers for both MS1 and MS2

MS levels	Parameters		Values
MS1	MS Scan Range		70~1000 m/z
	MS Resolution		70,000
	AGC target		1×10^6
	Maximum IT		200 ms
	Capillary temperature		350℃
	Sheath Gas flow		55 arb
	Aux Gas flow		10 arb
MS2	MS level(s)	Parameters	Values
	Full MS	Resolution	70,000 (DDA)<br>35,000 (DIA)
		AGC target	3×10^6 (DDA)<br>1×10^6 (DIA)
		Maximum IT	200 ms (DDA)<br>100 ms (DIA)
		Scan Range	70~1000 m/z
	MS2-Settings	Resolution	17,500
		AGC target	2×10^5
		Maximum IT	50 ms (DDA)<br>auto (DIA)
		Loop Count	10 (DDA)<br>7+3 (DIA of HILIC)<br>8+2 (DIA of C18)
		TopN	10 (DDA)
		Isolation Window	1.0 (DDA)
		Scan range	200~2,000
		(N)CE	15, 30, 45
		Minimum AGC target	1×10^2 (Targeted DDA of HILIC)<br>8×10^3 (Untargeted DDA)<br>1×10^2 (Targeted DDA of C18 Negative)<br>2×10^2 (Targeted DDA of C18 Positive)
		Intensity threshold	2×10^3 (Targeted DDA of HILIC)<br>1.6×10^5 (Untargeted DDA)<br>2×10^3 (Targeted DDA of C18 Negative)<br>4×10^3 (Targeted DDA of C18 Positive)

Supplementary Table 11. Design of SWATH-DIA for different modes for blood Samples.

Mode	MS levels	MZ Starting	MZ Ending	Scan Time/ms	Cycle Duration/ms
C18 ESI+	Full MS1	69.5	1000.5	140	~900
	SWATH	69.5	140.5	75	
	SWATH	139.5	210.5		
	SWATH	209.5	280.5		
	SWATH	279.5	350.5		
	SWATH	349.5	420.5		
	SWATH	419.5	490.5		
	SWATH	489.5	560.5		
	SWATH	559.5	630.5		
	SWATH	629.5	700.5		
	SWATH	699.5	1000.5		
C18 ESI-	Full MS1	69.5	1000.5	140	~900
	SWATH	69.5	140.5	75	
	SWATH	139.5	210.5		
	SWATH	209.5	280.5		
	SWATH	279.5	350.5		
	SWATH	349.5	420.5		
	SWATH	419.5	490.5		
	SWATH	489.5	560.5		
	SWATH	559.5	630.5		
	SWATH	629.5	750.5		
	SWATH	749.5	1000.5		
HILIC ESI+	Full MS1	69.5	1000.5	140	~900
	SWATH	69.5	130.5	75	
	SWATH	129.5	190.5		
	SWATH	189.5	250.5		
	SWATH	249.5	310.5		
	SWATH	309.5	410.5		
	SWATH	409.5	510.5		
	SWATH	509.5	610.5		
	SWATH	609.5	710.5		
	SWATH	709.5	810.5		
	SWATH	809.5	1000.5		
HILIC ESI-	Full MS1	69.5	1000.5	140	~900
	SWATH	69.5	135.5	75	
	SWATH	134.5	175.5		
	SWATH	174.5	215.5		
	SWATH	214.5	255.5		
	SWATH	254.5	295.5		
	SWATH	294.5	335.5		
	SWATH	334.5	375.5		
	SWATH	374.5	500.5		
	SWATH	499.5	750.5		
	SWATH	749.5	1000.5		

Supplementary Table 12. Summary of downloaded public MS2 libraries. The "-" indicates no version number available.

Database	URL	Version	Access Date
HMDB	https://hmdb.ca/downloads	5.0	Aug 14th, 2022
MoNA	https://mona.fiehnlab.ucdavis.edu/downloads	-*	Aug 15th, 2022
LipidBlast	https://mona.fiehnlab.ucdavis.edu/downloads	-	Aug 15th, 2022
MassBank	https://github.com/MassBank/MassBank-data/releases/latest	2022.06	Aug 19th, 2022
GNPS	https://gnps-external.ucsd.edu/gnpslibrary	-	Aug 30th, 2022
MINEs	https://minedatabase.mcs.anl.gov/#/download	-	Sep 15th, 2022
KEGG	https://rest.kegg.jp/	-	Aug 21st, 2022
LIPIDMAPS	https://www.lipidmaps.org/databases/lmsd/download	-	Feb 7th, 2023
LipidBank	https://lipidbank.jp/	-	Feb 7th, 2023
MiMeDB	https://mimedb.org/downloads	v1	Jun 29th, 2023
T3DB	http://www.t3db.ca/downloads	-	Jun 29th, 2023
FooDB	https://www.foodb.ca/downloads	v1.0	Jun 30th, 2023
Phenol-Explorer	http://phenol-explorer.eu/downloads	v3.6	Jun 30th, 2023
Exposome-Explorer	http://exposome-explorer.iarc.fr/downloads	-	Jul 1st, 2023
NORMAN Suspect List Exchange	https://www.norman-network.com/?q=suspect-list-exchange	-	Jul 1st, 2023
