# Supplementary material for: MetaboAnalystR 4.0: a unified LC-MS workflow for global metabolomics
Source: Nat Commun. 2024 May 1;15:3675. doi: 10.1038/s41467-024-48009-6 (PMC11063062; doi:10.1038/s41467-024-48009-6)
Supplement: Supplementary file 2 — Reporting Summary [file 41467_2024_48009_MOESM2_ESM.pdf]

nature portfolio

Corresponding author(s): Jianguo Xia

Last updated by author(s): Apr 8, 2024

Reporting Summary

Nature Portfolio wishes to improve the reproducibility of the work that we publish. This form provides structure for consistency and transparency in reporting. For further information on Nature Portfolio policies, see our Editorial Policies and the Editorial Policy Checklist.

Statistics

For all statistical analyses, confirm that the following items are present in the figure legend, table legend, main text, or Methods section.

n/a	Confirmed	
☐	☒	The exact sample size (*n*) for each experimental group/condition, given as a discrete number and unit of measurement
☐	☒	A statement on whether measurements were taken from distinct samples or whether the same sample was measured repeatedly
☐	☒	The statistical test(s) used AND whether they are one- or two-sided<br>*Only common tests should be described solely by name; describe more complex techniques in the Methods section.*
☐	☒	A description of all covariates tested
☐	☒	A description of any assumptions or corrections, such as tests of normality and adjustment for multiple comparisons
☐	☒	A full description of the statistical parameters including central tendency (e.g. means) or other basic estimates (e.g. regression coefficient) AND variation (e.g. standard deviation) or associated estimates of uncertainty (e.g. confidence intervals)
☐	☒	For null hypothesis testing, the test statistic (e.g. *F*, *t*, *r*) with confidence intervals, effect sizes, degrees of freedom and *P* value noted<br>*Give P values as exact values whenever suitable.*
☒	☐	For Bayesian analysis, information on the choice of priors and Markov chain Monte Carlo settings
☒	☐	For hierarchical and complex designs, identification of the appropriate level for tests and full reporting of outcomes
☒	☐	Estimates of effect sizes (e.g. Cohen's *d*, Pearson's *r*), indicating how they were calculated

Our web collection on statistics for biologists contains articles on many of the points above.

Software and code

Policy information about availability of computer code

Data collection	Thermo Scientific Xcalibur, version 4.3.73.11 was used for mass spectrometry data acquisition
Data analysis	MetaboAnalystR 4.0 (https://github.com/xia-lab/MetaboAnalystR); MS-DIAL 4.9.22; MS-FINDER 3.52; MZmine 3.2.8; XCMS 3.20.0; R 4.2.3; SIRIUS 5.6.3; KEGGREST 1.38.0; SLURM 22.05.6; Code used to analyze data and benchmarking studies are available from: https://github.com/Zhiqiang-PANG/MetabR4_scripts

For manuscripts utilizing custom algorithms or software that are central to the research but not yet described in published literature, software must be made available to editors and reviewers. We strongly encourage code deposition in a community repository (e.g. GitHub). See the Nature Portfolio guidelines for submitting code & software for further information.

Data

Policy information about availability of data

All manuscripts must include a data availability statement. This statement should provide the following information, where applicable:

- Accession codes, unique identifiers, or web links for publicly available datasets
- A description of any restrictions on data availability
- For clinical datasets or third party data, please ensure that the statement adheres to our policy

LC-MS and MS2 data of blood samples and serial dilutions used in the evaluation of MetaboAnalystR 4.0 have been uploaded to MetabolomicsWorkbench repository as studies ST002796 [https://metabolomicsworkbench.org/data/DRCCMetadata.php?Mode=Study&StudyID=ST002796] and ST002798 [https://metabolomicsworkbench.org/data/DRCCMetadata.php?Mode=Study&StudyID=ST002798] (https://www.metabolomicsworkbench.org/). The complex standards

mixture data was obtained from MetaboLights repository (ID: MTBLS2207 [https://www.ebi.ac.uk/metabolights/MTBLS2207]). The simple standard mixtures series data was from Curatr (https://curatr.mcf.embl.de/). The standard mixtures with medium complexity were also from MetaboLights repository (ID: MTBLS1311 [https://www.ebi.ac.uk/metabolights/MTBLS1311]). Polar and non-polar DDA metabolomics datasets of COVID-19 were downloaded from MetaboLights repository (ID: MTBLS2542 [https://www.ebi.ac.uk/metabolights/MTBLS2542]). The COVID-19 SWATH-DIA metabolomics dataset was obtained from MassIVE repository (ID: MSV000089568 [https://doi.org/doi:10.25345/C5HQ3S30D]). All curated MS2 spectral libraries and descriptions are available from the MetaboAnalyst website (https://www.metaboanalyst.ca/docs/Databases.xhtml).
KEGG database was accessed with KEGGREST (https://bioconductor.org/packages/release/bioc/html/KEGGREST.html).
HMDB: https://hmdb.ca/downloads
MoNA: https://mona.fiehnlab.ucdavis.edu/downloads
LipidBlast: https://mona.fiehnlab.ucdavis.edu/downloads
MassBank : https://github.com/MassBank/MassBank-data/releases/latest
GNPS: https://gnps-external.ucsd.edu/gnpslibrary
MINEs: https://minedatabase.mcs.anl.gov/#/download
KEGG : https://rest.kegg.jp/
LIPIDMAPS: https://www.lipidmaps.org/databases/lmsd/download
LipidBank : https://lipidbank.jp/
MiMeDB : https://mimedb.org/downloads
T3DB http://www.t3db.ca/downloads
FooDB https://www.foodb.ca/downloads
Phenol-Explorer http://phenol-explorer.eu/downloads
Exposome-Explorer http://exposome-explorer.iarc.fr/downloads
NORMAN Suspect List Exchange https://www.norman-network.com/?q=suspect-list-exchange

Research involving human participants, their data, or biological material

Policy information about studies with human participants or human data. See also policy information about sex, gender (identity/presentation), and sexual orientation and race, ethnicity and racism.

Reporting on sex and gender	N/A
Reporting on race, ethnicity, or other socially relevant groupings	N/A
Population characteristics	The human participants included in this manuscript for the blood sample dataset are paired. The participants in the study are adult university students without any diseases. All covariate-relevant factors of the participants, e.g. age were not contributing to the observational indicator.
Recruitment	Approval from the McGill University Institutional Review Board (#A05-M26-16B) was received fro this work. Whole blood (venous and capillary) was collected from consenting individuals from Mary Emily Clinic at McGill University in December 2016.
Ethics oversight	This study was approved by Research Ethics Office of McGill University (Study ID: A05-M26-16B).

Note that full information on the approval of the study protocol must also be provided in the manuscript.

Field-specific reporting

Please select the one below that is the best fit for your research. If you are not sure, read the appropriate sections before making your selection.

- [x] Life sciences
- [ ] Behavioural & social sciences
- [ ] Ecological, evolutionary & environmental sciences

For a reference copy of the document with all sections, see nature.com/documents/nr-reporting-summary-flat.pdf

Life sciences study design

All studies must disclose on these points even when the disclosure is negative.

Sample size	The sample size for whole blood sample study is 48, including 6 QC samples, and three groups (14 biological replicates for each group). The sample size is chosen based on the statistical analysis to make it enough for a confidence report. Usually, for both univariate and multivariate analysis, at least 6 samples per group is needed to reach the statistical meaning. Here, in the present study, we use far more samples to further ensure the effectiveness of statistical analysis.
Data exclusions	No data was excluded from the study.
Replication	Both LC-MS experiments and data processing have been repeated - 6 QC samples, and three groups (14 biological replicates for each group)
Randomization	Allocation into the experimental groups was random.

Blinding | Since there is only one group in the whole blood samples, the investigator processed the samples sequentially. There is no need for blinding. The data analysis is the most critical part, the bioinformatician was blinded to the data labels.

Reporting for specific materials, systems and methods

We require information from authors about some types of materials, experimental systems and methods used in many studies. Here, indicate whether each material, system or method listed is relevant to your study. If you are not sure if a list item applies to your research, read the appropriate section before selecting a response.

Materials & experimental systems

n/a	Involved in the study
☒	☐ Antibodies
☒	☐ Eukaryotic cell lines
☒	☐ Palaeontology and archaeology
☒	☐ Animals and other organisms
☒	☐ Clinical data
☒	☐ Dual use research of concern
☒	☐ Plants

Methods

n/a	Involved in the study
☒	☐ ChIP-seq
☒	☐ Flow cytometry
☒	☐ MRI-based neuroimaging

Plants

Seed stocks | *Report on the source of all seed stocks or other plant material used. If applicable, state the seed stock centre and catalogue number. If plant specimens were collected from the field, describe the collection location, date and sampling procedures.*

Novel plant genotypes | *Describe the methods by which all novel plant genotypes were produced. This includes those generated by transgenic approaches, gene editing, chemical/radiation-based mutagenesis and hybridization. For transgenic lines, describe the transformation method, the number of independent lines analyzed and the generation upon which experiments were performed. For gene-edited lines, describe the editor used, the endogenous sequence targeted for editing, the targeting guide RNA sequence (if applicable) and how the editor was applied.*

Authentication | *Describe any authentication procedures for each seed stock used or novel genotype generated. Describe any experiments used to assess the effect of a mutation and, where applicable, how potential secondary effects (e.g. second site T-DNA insertions, mosiacism, off-target gene editing) were examined.*
